# Automatic Prediction of Band Gaps of Inorganic Materials Using a Gradient Boosted and Statistical Feature Selection Workflow

**DOI:** 10.1021/acs.jcim.3c01897

**Published:** 2024-02-06

**Authors:** Son Gyo Jung, Guwon Jung, Jacqueline M. Cole

**Affiliations:** † Cavendish Laboratory, Department of Physics, University of Cambridge, J. J. Thomson Avenue, Cambridge CB3 0HE, U.K.; ‡ ISIS Neutron and Muon Source, STFC Rutherford Appleton Laboratory, Harwell Science and Innovation Campus, Didcot, Oxfordshire OX11 0QX, U.K.; § Research Complex at Harwell, Rutherford Appleton Laboratory, Harwell Science and Innovation Campus, Didcot, Oxfordshire OX11 0FA, U.K.; ∥ Scientific Computing Department, STFC Rutherford Appleton Laboratory, Harwell Science and Innovation Campus, Didcot, Oxfordshire OX11 0QX, U.K.

## Abstract

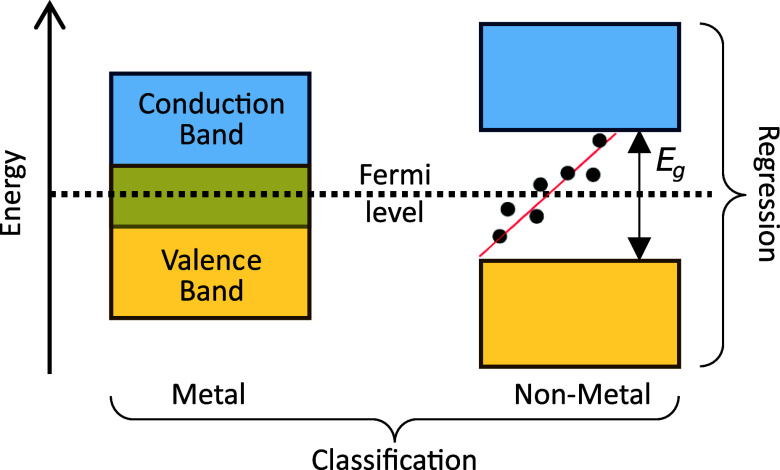

Machine learning (ML) methods can train a model to predict material properties by exploiting patterns in materials databases that arise from structure–property relationships. However, the importance of ML-based feature analysis and selection is often neglected when creating such models. Such analysis and selection are especially important when dealing with multifidelity data because they afford a complex feature space. This work shows how a gradient-boosted statistical feature-selection workflow can be used to train predictive models that classify materials by their metallicity and predict their band gap against experimental measurements, as well as computational data that are derived from electronic-structure calculations. These models are fine-tuned via Bayesian optimization, using solely the features that are derived from chemical compositions of the materials data. We test these models against experimental, computational, and a combination of experimental and computational data. We find that the multifidelity modeling option can reduce the number of features required to train a model. The performance of our workflow is benchmarked against state-of-the-art algorithms, the results of which demonstrate that our approach is either comparable to or superior to them. The classification model realized an accuracy score of 0.943, a macro-averaged F1-score of 0.940, area under the curve of the receiver operating characteristic curve of 0.985, and an average precision of 0.977, while the regression model achieved a mean absolute error of 0.246, a root-mean squared error of 0.402, and *R*^2^ of 0.937. This illustrates the efficacy of our modeling approach and highlights the importance of thorough feature analysis and judicious selection over a “black-box” approach to feature engineering in ML-based modeling.

## 1 Introduction

The analysis of band gaps (*E*_g_) of functional inorganic materials is pivotal to the design of many applications, including light-emitting diodes, photovoltaic cells, and transistors.^1−8^ There are well-established *ab initio* approaches that are used to predict *E*_g_. Theoretical methods, such as high-throughput electronic-structure calculations based on density functional theory (DFT), have played a vital role in accelerating the discovery of novel chemical materials in these fields of research.^9−17^ The process characterizing materials and their band gap properties has been streamlined via *ab initio* methods that facilitate computational simulations of material properties. This advancement has accelerated the exploration of diverse chemical landscapes across multiple research fields, a pace unattainable through conventional design-to-device processes.

While DFT calculations offer significant capabilities, they are often inaccurate or are not general enough, owing to inherent errors; these stem from their approximate nature and their requirement of additional chemical information that is neither standardized nor is readily available.^18−21^ A notable systematic discrepancy is observed in DFT-based calculations of *E*_g_, in that they frequently underestimate *E*_g_ relative to their cognate experimental values. These errors are attributed to approximations in the exchange–correlation functional and a derivative discontinuity term in the true density functional. Improved calculations for *E*_g_ can be afforded using hybrid functionals and GW-type methods.^22−24^ Yet, their high computational cost makes them unsuitable for rapid chemical property predictions. Additional functionals exist that can afford the accurate prediction of *E*_g_ without such an increase in the computational requirement. These include the Becke-Johnson (mBJ) potential and the generalization of Δ-self-consistent field (Δ-SCF) to solids.^25−27^ However, there are limitations associated with them. For instance, the mBJ functional is highly effective for many semiconductors and insulators, but it struggles with ferromagnetic metals, while the Δ-SCF method relies on the dielectric screening properties of electrons.^27−29^ It is also important to highlight that DFT calculations are mostly restricted to ordered crystal structures, and their accuracy falters for highly correlated systems. However, the integration of DFT + U can ameliorate such limitations.^30^

These efforts within computational materials science have led to the creation of data repositories with extensive sets of computed chemical structures and their properties, such as the Materials Project (MP).^31,32^ The accessibility of these chemical data, coupled with the rise of big-data initiatives, has resulted in a growing interest in data-driven methods owing to their proficiency in processing and analyzing large-scale, high-dimensional data sets.

In materials science, data-driven approaches leverage materials informatics and machine learning (ML),^33−37^ which may include electronic structure calculations. A typical materials informatics workflow involves transforming the *ab initio* chemical information into a machine-readable format using feature descriptors.^38−43^ The generated features are then used for model training, which facilitates the statistical prediction of: (i) properties of unseen chemical materials in a regression problem or (ii) the specific class or category the materials are associated with in a classification problem. The rationale is to empower ML models to deduce chemical structure–property relationships that exceed the capabilities of manual analysis. These techniques have showcased their prowess in accurately predicting chemical structures and properties, including the use of a multifidelity modeling strategy that harnesses both DFT calculations and experimental measurements.^44−46^ This exemplifies the effectiveness of materials screening for the realization of novel materials within highly complex feature spaces for various applications.

Various ML techniques have been employed in the prediction of *E*_g_ against the DFT calculations. For instance, Gladkikh et al.^47^ demonstrated the use of kernel ridge regression (KRR), extremely randomized trees, and alternating conditional expectations to predict *E*_g_ of ABX3 perovskites from elemental properties. Pilania et al.^48^ leveraged a KRR model to estimate *E*_g_ for double perovskites. Pilania et al.^29^ also explored a multifidelity ML modeling approach, where a multifidelity cokriging statistical learning framework is used to amalgamate variable-fidelity quantum mechanical calculations, to generate an ML model based on a Gaussian process regression. A support vector regression (SVR) with a radial kernel was used by Huang et al.^49^ to predict both the band offset and *E*_g_ of nitride-based semiconductors. Similarly, Lee et al.^50^ employed SVR for the prediction of *E*_g_ of inorganic compounds. Other approaches include the use of crystal graph convolutional neural networks^51^ and gradient boosting decision trees (GBDTs).^52^ These studies demonstrate the applicability of ML in computational material science. However, the models in these studies had been trained on *E*_g_ values that were derived from diverse DFT calculations using different functionals. Considering the inherent imprecision of these calculations, achieving a close alignment with experimental values poses a challenge for the models unless additional adjustments or corrections are made, which, in turn, will incur a high computational cost.

There have also been efforts to develop ML-based solutions to predict *E*_g_ against experimental measurements. An artificial neural network-based solution was proposed by Zhaochun et al.^53^ to predict *E*_g_ and the melting point of binary and ternary compound semiconductors. An SVR technique was employed by Gu et al.^54^ for the prediction of *E*_g_ of binary and ternary compound semiconductors using a set of experimental data, consisting of 25 binary compounds and 31 ternary compounds. Other regression approaches were explored, which involved ordinary least-squares (OLS) or least absolute shrinkage and selection operator (Lasso), or even the elastic net, which linearly combines the penalties of the Lasso and ridge regression methods.^55^ One notable study is by Zhou et al.,^56^ who trained a support vector classification and a SVR on experimental measurements to classify and predict *E*_g_ of inorganic solids; this data set has become a part of the Matbench test suite v0.1.^57^ Zhou et al. trained these models using 136 features, or variables, that were generated solely from the chemical composition of a material. This meant that only chemical composition is required to compute an estimate of *E*_g_ against experimental values. When examining the area under the curve of the receiver operating characteristic curve (AUC-ROC), they realized an AUC-ROC of 0.97 for the classification of materials by metallicity, while a coefficient of determination (*R*^2^) of 0.90 and a root-mean squared error (RMSE) of 0.45 eV were achieved for the regression analysis of *E*_g_ against experimental measurements. Other algorithms have been applied to such a benchmark set to evaluate their efficacy. The range of mean absolute errors (MAE) realized using alternative algorithms is 0.3310–0.4461 eV.^57−63^ See Section 2 Methods for the definition of these performance metrics.

Various studies within this domain showcase a diverse range of methodologies. While certain studies depend upon a restricted set of experimental data, others harness sophisticated algorithms, frequently incorporating a substantial number of input features to attain the previously mentioned model performance. In general, there is a noticeable deficiency in addressing comprehensive statistical feature analysis and selection, mitigating multicollinearity, and conducting permutation analysis, among other considerations. Moreover, exploration of optimization strategies aimed at enhancing model generalization appears to be inadequately discussed.

In this study, we employ the gradient boosted and statistical feature selection (GBFS) workflow, which we have designed for materials-property predictions.^44^ The GBFS workflow integrates a distributed gradient boosting framework, in conjunction with exploratory data and statistical analyses and multicollinearity treatments, to discern a subset of features that are highly relevant to the target variable or class within a complex feature space; this affords minimal feature redundancy and maximal relevance to the target variable or classes. The efficacy and the efficiency of the workflow has been showcased in previous materials-property predictions against DFT calculations.^44^

Here, we extend our research into the domain of prediction, utilizing experimental data. Specifically, we implement the GBFS workflow to predict *E*_g_ against experimental measurements and explore a multifidelity modeling strategy by augmenting these experimental data with DFT calculations from the MP. Our objective is to showcase the versatility of the proposed workflow as a general-purpose tool, extending beyond the confines of specific data types such as DFT calculations. Additionally, we sought to comprehend the impact of enhancing the predictive model by incorporating data from various streams. The performance of our models is compared to state-of-the-art reports from the literature.

For a like-for-like comparison to the work of Zhou et al., we confine our descriptor sets to those based on chemical composition alone, understanding that, most typically, experimental *E*_g_ records in the literature lack comprehensive crystallographic information. Later, we extend our analysis to another set of experimental measurements, namely, by Kiselyova et al.^64^ Our method highlights the importance of thorough feature analysis and judicious selection over merely complex modeling, as a simple tree-based model trained on features selected and engineered by the GBFS workflow can yield results that are comparable or superior to those reported in the literature. The workflow additionally provides insights into feature interactions and their relevance to the target variable. Furthermore, we apply our final classification and regression models to chemical compositions in Pearson’s Crystal Structure Database (94,095) and the MP (105,583). The results are made available as a part of the Supporting Information that serves as a resource for researchers in inorganic material design.

## 2 Methods

The experimental measurements employed in this study were compiled from diverse literature sources, as referenced.^56,64−67^ The 154,718 DFT calculations utilized for the multifidelity modeling were obtained from MP.^31,32^ The results presented in Sections 3.1 and 3.2 were generated using a data set of 6354 chemical compositions. This is identical to the data set utilized by Zhou et al.,^56^ facilitating a like-for-like comparison. The additional regression analysis in Section 3.3 considers 7588 chemical compositions, primarily from the work of Kiselyova et al.^64^

**Figure 1:**
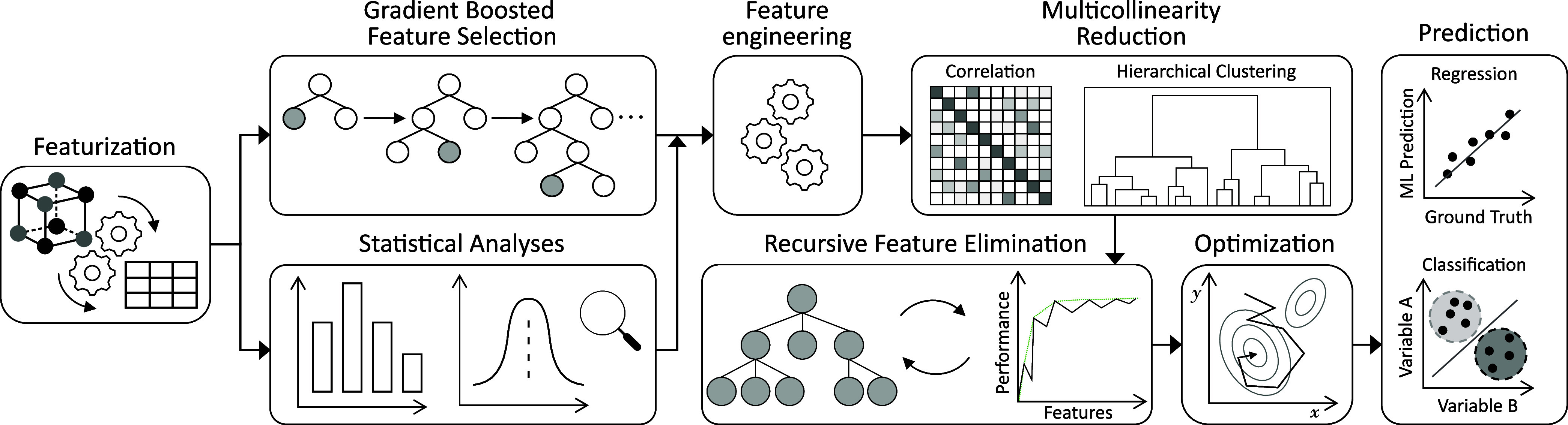
Overview of our operational workflow as described in Section 2—Methods. See ref (44) for a more detailed description.

A high-dimensional feature vector was generated by leveraging a set of composition-based descriptors. These include the composition featurizer modules from Matminer^68^ and Pymatgen.^69^ Further features were created using statistics taken over elemental attributes for a particular chemical composition. These calculations are based on data sources, which includes Magpie,^61^ Pymatgen,^69^ Deml,^70^ and the neural network embeddings of elements generated using the MatErials Graph Network.^71^ Moreover, the GBFS workflow combines the following: (i) a gradient boosting framework to determine a subset of features that maximize their relevance to the target variable or class, (ii) statistical analyses of the exploratory features to identify those statistically significant to the target variable or class, (iii) a feature engineering step to generate additional features, (iv) a two-step multicollinearity reduction to obtain minimal feature redundancy, which involves a correlation and hierarchical cluster analyses, (v) a recursive feature elimination (RFE), and (vi) a Bayesian optimization to determine the architecture of the final predictive ML model. See Figure 1 for the schematic diagram of the workflow. More details of the methodological workflow can be found in ref (44).

For the classification analysis, we consider the accuracy and F1-score, where the latter is defined as the harmonic mean of the precision and recall as follows
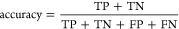
1
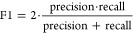
2
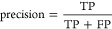
3
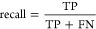
4where TP and TN are true positive and true negative, and FP and FN are false positive and false negative, respectively. For the regression analysis, we consider the MAE, the mean squared error (MSE), and the coefficient of determination that is defined as the square of the Pearson correlation coefficient, *R*
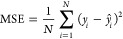
5
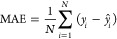
6
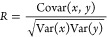
7where *y* and *ŷ* are the true and predicted values, respectively, over *N* number of samples;  is the covariance between *x* and *y*;  and  are the variance of *x* and *y*;  and  are the mean of *x* and *y*, respectively. The range of *R* is [−1, 1], and its value indicates the linear tendency of a quantity to change as the values of another is varied.

## 3 Results and Discussion

### 3.1 Classification of Materials by Metallicity

#### 3.1.1 Performance Results

**Figure 2:**
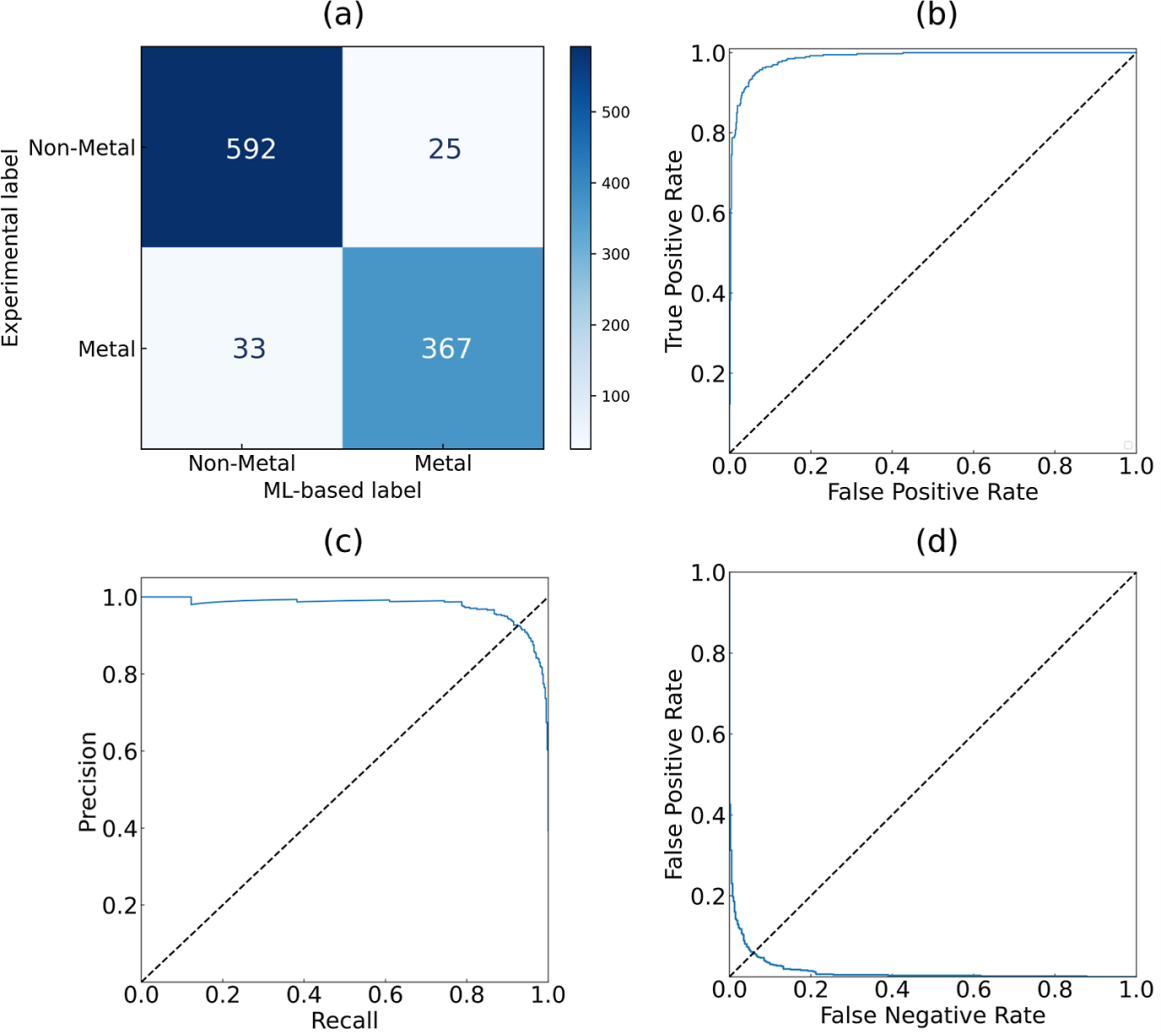
Classification performance on the test set. (a) Confusion matrix, (b) ROC, (c) PR, and (d) DET curves. The macro-averaged AUC-ROC of 0.985, AP of 0.977, and EER of 6% were achieved.

The classification of materials by their metallicity was performed by using a gradient boosting algorithm using 27 (out of 827) features selected via the GBFS workflow. The model performance is illustrated in Figure 2, along with the performance metrics that are summarized in Table 1. An accuracy score (eq 1) of 0.943 and a balanced accuracy score of 0.938 were achieved. The ROC curve illustrates a diagnostic ability of the model toward the target classes, as the classification threshold is varied by depicting the variation of the true positive rate against the false positive rate (FPR). A macro-averaged AUC-ROC of 0.985 was realized. This indicates that the final classifier is highly discriminative toward the two target classes; this is consistent with the output class-probability distribution (in Supporting Information 1), which is illustrative of an almost binary outcome. In comparison, Zhou et al. achieved an accuracy of 0.92 and an AUC-ROC of 0.97.

**Table 1:** Summary of the Performance Metrics for the Final Classifier When Applied to the Test Set

	precision	recall	F1-score
metal	0.947	0.959	0.953
nonmetal	0.936	0.917	0.927
macro average	0.942	0.938	0.940
weighted average	0.943	0.943	0.943

The detection error trade-off (DET) shows the change in the FPR relative to the false negative rate (FNR) as the classification threshold is varied. The point at which the FPR and FNR cross the diagonal, known as the equal error rate (EER), is approximately 0.06. This result suggests that a low error rate can be obtained in the model predictions when using selected features. The fact that the plot profiles are fairly symmetric about their respective diagonals appears to indicate that the class imbalance has been well corrected. Moreover, the precision–recall (PR) curve was plotted to assess the effectiveness of the model, owing to the presence of an imbalanced data set. The PR curve demonstrates the quality of the model performance by illustrating the trade-off between precision and recall as the classification threshold is varied. This is important because imbalanced classes can lead to the FPR becoming less informative as a large number of negatives (i.e., false positives and true negatives) yields a low FPR, while still allowing poor precision. An average precision (AP) of 0.977 was achieved; this is the average of the precision at each classification threshold that is weighted by the change in the recall from the prior threshold. The result indicates that both high precision and recall are realized, where a low FPR and a low FNR are attained. This implies that the model is capable of returning a high percentage of positives (i.e., true positives and false negatives) that are mostly classified correctly.

The overall model performance can be evaluated via the F1-score (see eqs 2–4). The macro- and weighted-averaged F1-scores were 0.940 and 0.943, respectively. Zhou et al. did not state their F1-score. The benchmark scores from the Matbench test suite v0.1 show the highest F1-score and balanced accuracy score as 0.920 and 0.921, respectively. This further supports that our classifier is highly discriminative toward the two target classes.

Upon closer examination of the predictions, clear trends are discernible in the characteristics of misclassified chemical compounds. An analysis of the two most influential features, based on the total loss reduction, reveals distributions that differ from those observed in the training set. In the scaled distribution of the one-hot-encoding of the highest occupied molecular orbital (HOMO) character corresponding to the p-orbital, we observed that in the training set, the average feature values for nonmetals and metals are ca. 0.82 and 0.26, respectively. This finding indicates a stronger association of nonmetals with HOMO with p-orbital characteristics. A similar feature distribution is noted among the correctly classified compounds in the test set. However, among misclassified compounds in the test set, the average feature values deviate to 0.40 for nonmetals and 0.67 for metals, which goes against the general trend.

Similar observations hold true when the mean number of filled valence p-orbitals among elements in the chemical composition. The scaled distributions between correctly classified chemical compounds and those in the training set are consistent, hovering around 0.50 and 0.26 for nonmetals and metals, respectively. This suggests that nonmetals tend to have a higher mean number of filled valence p-orbitals. In contrast, misclassified compounds exhibit feature values of ca. 0.46 for nonmetals and ca. and 0.42 for metals. Once more, this observation contradicts the overarching pattern. Notably, compounds misclassified as metals manifest a distribution that is closer to nonmetals in both cases. It seems that the model tends to classify chemical compounds as nonmetals when relatively higher feature values are observed for these two features, which are associated with p-orbitals.

These observations align with the fundamental principles of chemistry, as the majority of nonmetal and metalloid elements are situated in the p-block, encompassing groups 13–18 of the periodic table. The p-block comprises chemical elements in which np orbitals are filled, resulting in distinctive chemical properties that distinguish them from those of elements in other blocks of the periodic table. Consequently, understanding the statistical metrics related to the number of filled p-valence orbitals appears to offer insights into chemical elements that are highly discriminatory toward the target classes. This leads to specific chemical elements being associated with having a greater likelihood of being a nonmetal, particularly for elements such as Si ([Ne]3s^2^3p^2^), Ge ([Ar]4s^2^3d^10^4p^2^), and As ([Ar]4s^2^3d^10^4p^3^), which are common in semiconductors and belong to the p-block in periods 3 and 4 and in groups 14 and 15.

#### 3.1.2 Oversampling

The benchmark data set used to achieve the results above consists of 6354 compounds, which partition into 2458 metal (*E*_g_ > 0 eV) and 3896 nonmetal (*E*_g_ = 0 eV) materials, and a train-to-test split ratio of 4:1. A total of 827 composition-based features were computed (see Methods for more details). The imbalanced data set was treated by applying the smoothed random oversampling (smoothed-ROS) method with a shrinkage of 0.35, which is an extension of ROS with the introduction of Gaussian noise. The noise is stochastic. Therefore, it prevented an overtraining of the model on particular values of a feature due to the increased intraclass sample variability, and it appeared to improve model generalization. We found the smoothed-ROS method to be the most effective in alleviating potential learning biases among other oversampling techniques.

#### 3.1.3 Gradient Boosted and Statistical Feature Selection Workflow

**Figure 3:**
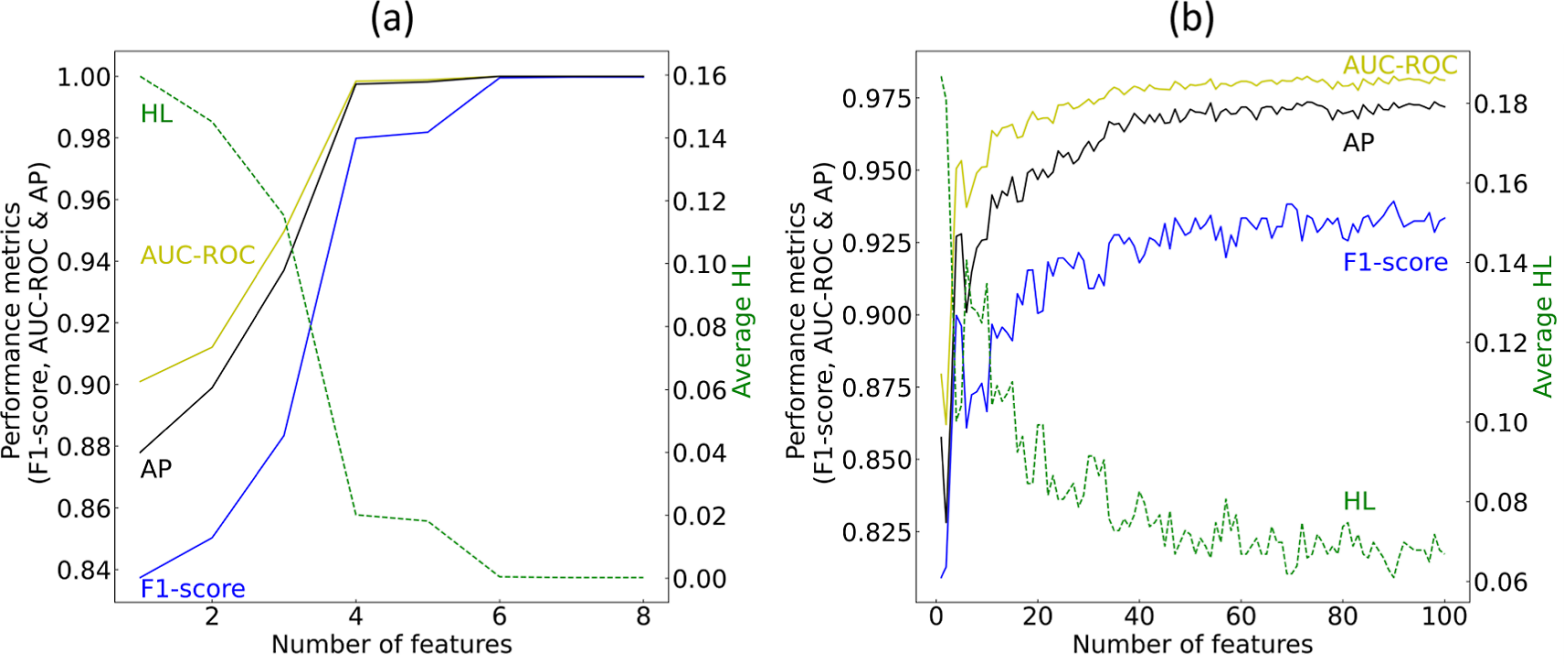
Gradient boosting feature selection results of the classification of materials by metallicity. Performance of GBDTs on (a) the training set and (b) the validation set, where classification models are trained recursively with an increasing subset of features, beginning from the most relevant feature based on the realized total loss reduction.

The recursive training of GBDTs with an increasing subset of features showed the convergence of AUC-ROC, AP, F1-score, and the average Hamming loss on the training set using ca. 8 of the most relevant features; the feature relevance ranking was initially obtained by observing the loss reduction achieved by each feature when training a GBDT with the entire features. On the validation set, convergence of all four performance metrics was observed once the first ca. 60 features were considered in the model training. The results are shown in Figure 3.

#### 3.1.4 Feature Analyses and Feature Engineering

With these 60 features selected, we performed a generalization of the one-way analysis of variance F-test, Pearson’s chi-squared test, mutual information (MI) analysis, and discriminant analysis using logistic regression. Examples of features found to be statistically significant were as follows: the maximum number of filled p-valence orbitals, the average number of p-valence electrons, the mean group of the periodic table, the thermal conductivity, the minimum coefficient of linear thermal expansion, the mean Mendeleev number, the HOMO energy, the mean melting point, and the one-hot-encoding of HOMO character corresponding to the p-orbital. These are consistent with our previous analysis,^44^ where a full discussion of the feature interaction and interpretation is made. The features selected by GBFS and statistical analyses were used to engineer new features via the brute-force method. This yielded an additional 159 new features, leading to a total of 219 features that comprised the preliminary subset of features for the classification analysis.

#### 3.1.5 Multicollinearity Reduction and Recursive Feature Elimination

Multicollinearity effects within the data set were reduced by removing features with a correlation coefficient of 0.8 or higher. This reduced the number of features to 105. Further treatment of multicollinearity effects was carried out via a hierarchical cluster analysis, which uses the Spearman rank-order correlation with 1.5 units of Ward’s linkage distance as the threshold; this led to the retention of 41 features since only a single feature from each cluster was kept, where the optimal distance threshold was identified using the Elbow method. The corresponding dendrogram of the hierarchical agglomerative clustering, which illustrates the formation of clusters moving up the dendrogram, and the 10-fold permutation feature-importance analysis can be found in Supporting Information 2. Subsequently, the optimal subset of features was identified by eliminating 14 further features via the 10-fold RFE, using a weighted F1-score as the performance metric (see Supporting Information 3 for the RFE plot). This resulted in the final subset of 27 features, identified from among 827 original and 159 engineered features, to be most relevant to the target classes without any prior knowledge of the domain. In contrast, Zhou et al. employed a total of 136 features in the final model.

#### 3.1.6 Model Optimization

A two-step optimization process was followed to determine the architecture of the final classifier. The hyperparameters of the model were optimized by using a combination of grid search and Bayesian optimization by using Gaussian processes. An initial hyperparameter tuning process was performed by scanning the hyperparameter space by using the grid-search method. This subsequently identified the region in which Bayesian optimization was to be applied. Such an optimization strategy proves to be particularly effective for an objective function that has no closed form and is expensive to evaluate and in cases when the evaluations result in noisy responses. The convergence, partial dependence, and evaluation plots from the Bayesian optimization results are shown in Supporting Information 4, and the total loss reduction (i.e., the feature-relevance ranking) realized by the final set of features is shown in Supporting Information 5. In general, the types of features that were selected for the final classification analysis are as anticipated. This finding is consistent with the results from the statistical analyses and those obtained using the SHapley Additive exPlanations (SHAP) framework,^72^ which is a game theoretic approach to explain the output of an ML model. See Supporting Information 6 for the average contribution and beeswarm plots from the SHAP analysis.

### 3.2 Regression Analysis of Band Gap

#### 3.2.1 Performance Results

**Figure 4:**
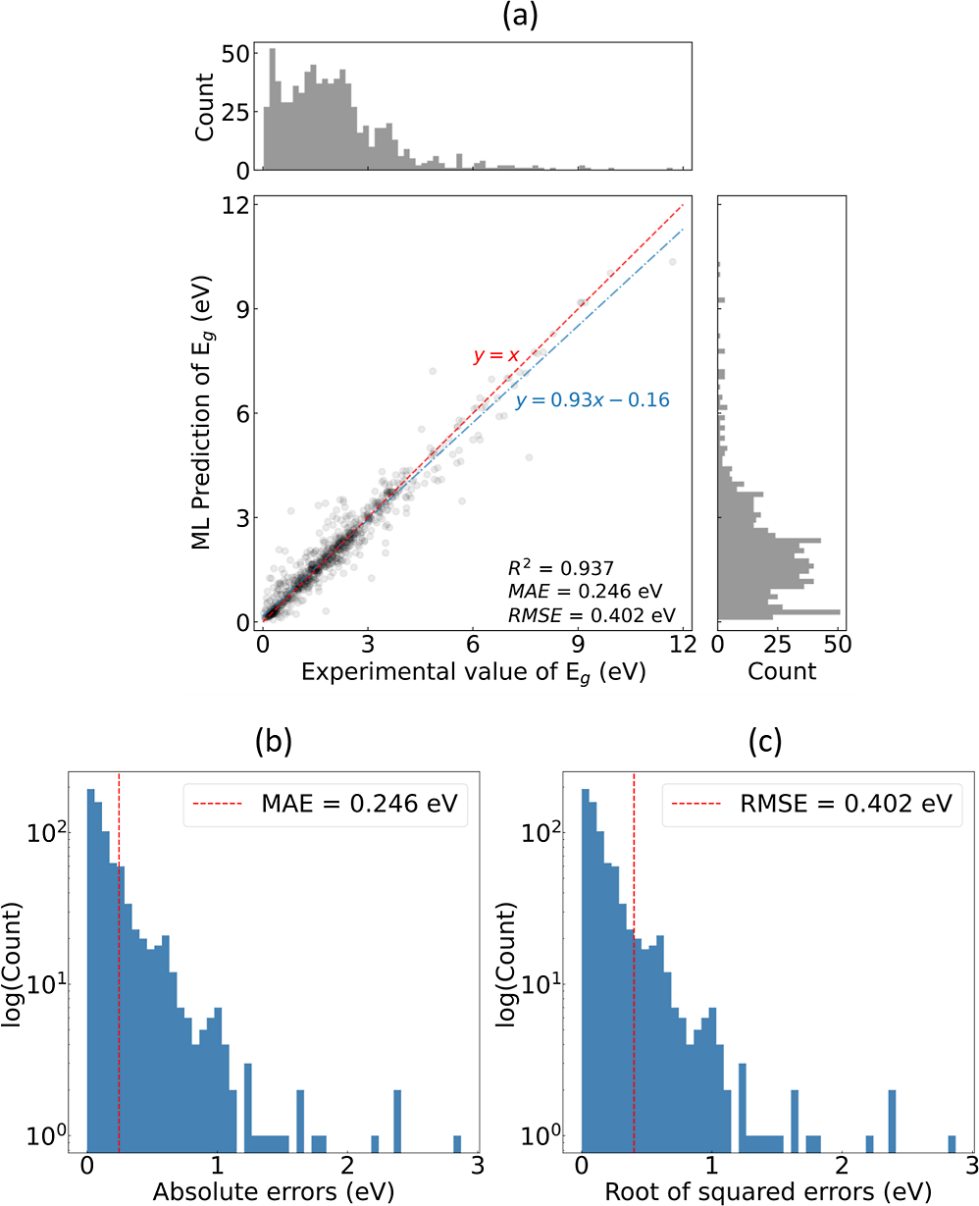
(a) Regression of the ML-based predictions of *E*_g_ against experimental measurements, where the regression models are trained on the final subset of features selected by the GBFS workflow. The dashed red line is drawn to represent the hypothetical case, where the ML-based prediction would equal the experimental measurement. The solid blue line is a linear fit generated using the OLS method. Distribution of the (b) absolute errors and (c) root of squared errors of the ML-based prediction of *E*_g_ are shown, where the dashed red line indicates the MAE and RMSE, respectively.

The regression analysis of *E*_g_ was performed using a gradient boosting algorithm with 46 features selected via the GBFS workflow. The model performance and error distributions are listed in Figure 4. The solid blue line is the line of best fit between the experimental measurements and ML-based predictions, generated using the OLS method. The linear fit has a gradient of 0.93 and a *y*-intercept of 0.16 eV. The *y*-intercept may indicate a small systematic bias for small values of *E*_g_. Nevertheless, the linear fit illustrates the close correspondence between the experimental measurements and ML-based predictions. This is further supported by the *R*^2^ value of 0.937, which indicates a high correlation between the prediction and the ground truth. Moreover, MAE and RMSE values of 0.246 and 0.402 eV were realized, respectively, where a greater degree of error in the latter is due to the greater penalization of predictions with larger deviations from their true values (see eqs 5–7). The distributions of absolute errors and root of squared errors are shown in Figure 4b,c, respectively. In comparison, Zhou et al. achieved an *R*^2^ of 0.90 and RMSE of 0.45 eV, which also demonstrates the robustness of their SVR model. Our model performance is comparable or superior to those achieved with alternative methods (cf. the aforementioned range of MAE in Introduction).

**Figure 5:**
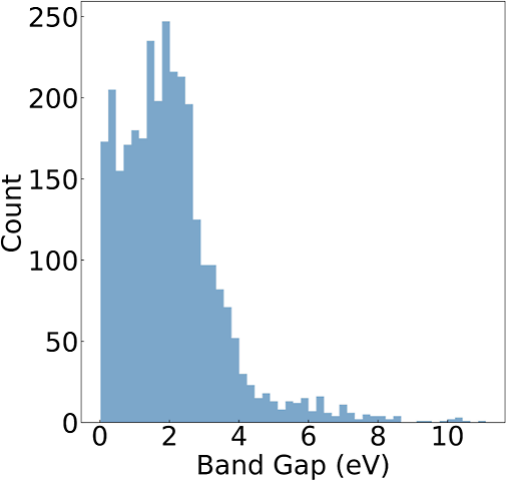
Distribution of band gap (*E*_g_) values of nonmetals in the training set.

A closer examination of the training set reveals that the chemical composition with the smallest *E*_g_ of 0.02 eV corresponds to Pb_0.87_Sn_0.13_Se and the largest *E*_g_ of 11.1 eV corresponds to MgF_2_. The *E*_g_ value is less than 3 eV for the majority of the chemical compositions of materials in the training set, as illustrated by the population distribution plot in Figure 5. This explains the relatively larger deviations in the prediction for semiconductors with larger *E*_g_ values. Specifically, we observe an underestimation of *E*_g_ where its value is large, as depicted by the line of best fit (in blue) in Figure 4a, which sits below the diagonal line in red. We attribute such an observation to the lack of wide or ultrawide band gap semiconductors in the training data. Nevertheless, the ML modeling approach has demonstrated an accurate prediction of *E*_g_ in the absence of structural information. This is despite the fact that our methodology does not involve specific adjustments or treatments to accommodate different crystal forms of the same chemical compound, a phenomenon known as polymorphism. Our approach strictly trains the model to be agnostic to polymorphs, aligning with the methodology employed by Zhou et al. This ensures a like-for-like comparison in our study. This is to say that the models demonstrate good performance, even though they are unaware of the different crystal forms that the sample compound may adopt, owing to the various ways in which chemical elements can arrange within unit cells of the crystalline lattice of each crystal form. See Section 3.3 for the additional regression analysis where we evaluate the model predictions against the median and mean experimental *E*_g_ values. Taking our attention back to the error distribution plots in Figure 4, which has a log scale in the *y*-axis, we see that the majority of predictions have an error below ca. 1 eV. At a closer examination, out of 780 chemical compositions in the test set, 670 predictions (ca. 86%) have an absolute error below 0.5 eV and 545 predictions (ca. 70%) have an absolute error below 0.25 eV.

The results discussed thus far present predictions of *E*_g_ values against experimental measurements with promising statistical figures-of-merit. Nonetheless, it is important to validate these results by considering how these predictions fare against well-studied inorganic compounds rather than simply demonstrating their collective statistical quality in an anonymized form. A comparative analysis is, therefore, conducted against six unseen compounds that are extensively researched both experimentally and at various theoretical levels. The results are summarized in Table 2.

**Table 2:** Summary of the Band Gap Predictions (*E*_g_ in eV) against Experimental Measurements

composition	experimental *E*_g_	GBFS *E*_g_	SVR *E*_g_(56)	PBE *E*_g_	GW *E*_g_	HSE *E*_g_
PbTe	0.19^73^	0.215 (13%)	0.2 (5%)	0 (−100%)^73^	0.26 (36%)^73^	0.19 (0%)^74^
CuSbS_2_	1.38^75^	1.40 (1%)	1.39 (1%)	0.9 (−35%)^75^	1.1 (−20%)^75^	1.69 (22%)^75^
GaN	3.2^73^	3.00 (−6%)	4.45 (39%)	1.62 (−49%)^73^	3.32 (4%)^73^	3.14 (−2%)^26^
TiO_2_	3.42^24^	3.25 (−5%)	3.99 (16%)	2.13 (−37%)^24^	3.73 (9%)^24^	3.67 (7%)^24^
ZnS	3.91^73^	3.41 (−13%)	3.12 (−20%)	2.07 (−47%)^73^	4.15 (6%)^73^	3.49 (−11%)^26^
LiF	14.2^73^	10.53 (−26%)	9.87 (−30%)	9.2 (−35%)^73^	15.1 (6%)^73^	11.47 (−19%)^76^
MAE		0.76	1.16	1.73	0.32	0.63
RMSE		2.29	3.54	5.47	0.18	1.30

The aforementioned underestimation of DFT-based *E*_g_ values that have been computed using a PBE functional is clearly apparent among the results, with a negative percentage difference being shown across all compounds. Although relatively smaller in magnitude, our model (labeled GBFS *E*_g_) exhibits a negative percentage difference beyond an *E*_g_ value of ca. 3 eV. This is consistent with our previous discussion in the regression analysis, where we observed a systematic positive bias of ca. 0.16 eV at low values of *E*_g_ and an underestimation at higher *E*_g_ values, with a gradient of 0.93 between the ML prediction and the ground truth—an effect that becomes exaggerated at higher values of *E*_g_. This explains the largest deviations that are observed, for example, in LiF, which predicts an *E*_g_ value of 14.2 eV. We attribute this anomaly to the lack of wide or ultrawide band gap semiconductors within the training set, as previously discussed. Nevertheless, ML-based predictions (both GBFS and SVR *E*_g_) yielded lower MAE and RMSE values when compared with high-throughput calculations that incorporated a PBE functional. The result pertaining to GW-type calculations realized the lowest errors among the methods. However, such an approach incurs the greatest computational cost and cannot provide an efficient and automated approach to the *E*_g_ prediction. The second lowest errors originated from the high-throughput calculations that employed hybrid functionals (HSE); this is another computationally expensive approach. The MAE achieved using HSE is comparable to the value obtained by our method, while the RMSE is ca. 1 eV lower for the former. Again, this difference stems from the error associated with wide and ultrawide band gap semiconductors. We anticipate a significant improvement of ML models pending the availability of a significantly greater number of ultrawide band gap semiconductors.

We now delve into a more detailed examination of the feature interactions that contributed to the aforementioned results in the regression analysis. The highest relevance, as indicated by the realized total loss reduction, is associated with the HOMO energy (feature number *m* = 593). This is succeeded by the standard deviation of the periodic group among elements in the chemical composition (*m* = 258), the fraction of p-valence electrons (*m* = 586), the average Mendeleev number among elements in the chemical composition (*m* = 115), the average deviation of electronegativity among elements in the chemical composition (*m* = 158), and the average deviation of the number of filled valence p-orbitals among elements in the composition (*m* = 170).

The selection of HOMO energy by the GBFS workflow as the feature with the highest relevance was anticipated given that it is a parameter directly involved in *E*_g_ estimation. The correlation between the HOMO energy and *E*_g_ is ca. −0.58 in the training data, indicating that lower HOMO energies correspond to larger *E*_g_ values. This correlation is logical as the HOMO represents the highest energy molecular orbital that contains electrons, akin to the valence band in Band theory. The energy of the lowest unoccupied molecular orbital represents the minimum energy level into which an electron can be excited. While its inclusion was expected, its correlation with the *E*_g_ values in the training data is not as pronounced as that of the HOMO energy, registering a correlation magnitude of only 0.17.

The subsequent set of features that are deemed significant in predicting *E*_g_ are associated with statistical values based on the period group number and Mendeleev order number among elements in the chemical composition. These features are directly linked to the chemical compositions of the compounds. The standard deviation of the periodic group among elements in the chemical composition exhibits a correlation of ca. 0.38 with the target variable. This suggests a discernible trend between the two, indicating that higher deviations in the periodic group number among elements are associated with elevated values of *E*_g_. This aligns with the observation that some of the largest *E*_g_ values in the training data are found in chemical compositions such as MgF_2_, NaF, RbF, and BeO. These compositions involve *s*-block metals in groups 1 and 2 paired with p-block nonmetals and halogens in groups 16 and 17 (e.g., oxygen and fluorine), generating some of the largest standard deviations of the periodic group among elements in the chemical composition. Moreover, the Mendeleev number, distinct from the atomic numbering system, is an ordering assigned to each chemical element in the periodic system. Its purpose is to arrange elements so that those with similar behaviors are placed consecutively. Similar to the previous feature, a clear trend is apparent, wherein a lower mean Mendeleev number among elements in the composition correlates with higher values of *E*_g_, with a correlation of ca. −0.53. In the training data, chemical compounds with some of the highest *E*_g_ values exhibit a mean Mendeleev number of ca. 10.

Another important set of features to be acknowledged involves p-valence electrons. Two relevant features are identified by the GBFS workflow, which are (i) the fraction of p-valence electrons and (ii) the average deviation of the number of filled valence p-orbitals among elements in the chemical composition. As discussed in the classification problem, these observations align with fundamental principles of chemistry, given that the majority of nonmetal and metalloid elements reside in the p-block, spanning groups 13–18 of the periodic table. The p-block encompasses chemical elements in which np orbitals are filled, resulting in distinctive chemical properties that set them apart from elements in other blocks of the periodic table. Notably, elements such as Si, Ge, and As, which are well-known in semiconductor applications, fall within this category. A correlation of 0.61 is observed against *E*_g_ values in the training data for the fraction of p-valence electrons.

The final among the most pertinent features to be discussed is the average deviation of electronegativity among elements in the chemical composition. This feature demonstrated the highest correlation with the *E*_g_ values in the training set, registering a value of ca. 0.68. The elements with the highest electronegativity are typically found in groups 16 and 17 (e.g., fluorine, oxygen, and chlorine), while those with the lowest electronegativity are situated in groups 1 and 2 (e.g., sodium, lithium, potassium, and magnesium). Consequently, the highest average deviation of electronegativity is generally observed between these two regions of the periodic table. The rationale behind selecting this feature lies in the fact that pairing a metal with a nonmetal element corresponds to a large difference in orbital energy. This phenomenon becomes more pronounced as the disparity in electronegativity between the pair of elements increases. In other words, a substantial difference in the electronegativity of two elements in a compound leads to an increase in its ionic properties, which in turn reduces the overlap of orbitals and elevates *E*_g_ of a material. Consequently, the probability of a material exhibiting a larger *E*_g_ increases when elements with higher electronegativity, concentrated in the p-block, are incorporated into the material composition. This is intuitive, as the strength of ionic bonding or electrostatic interaction is directly determined by the difference in electronegativity between neighboring ions. The result implies that nonmetal elements, such as oxygen and fluorine, are important attributes to consider when distinguishing metallic bonding from other types of bonding. This stands to reason since a metal can exist in the form of oxides, while ionic and metallic characteristics are distinct; an increase in the former contributes to an increase in *E*_g_ as orbital overlap decreases.

#### 3.2.2 Gradient Boosted and Statistical Feature Selection Workflow

**Figure 6:**
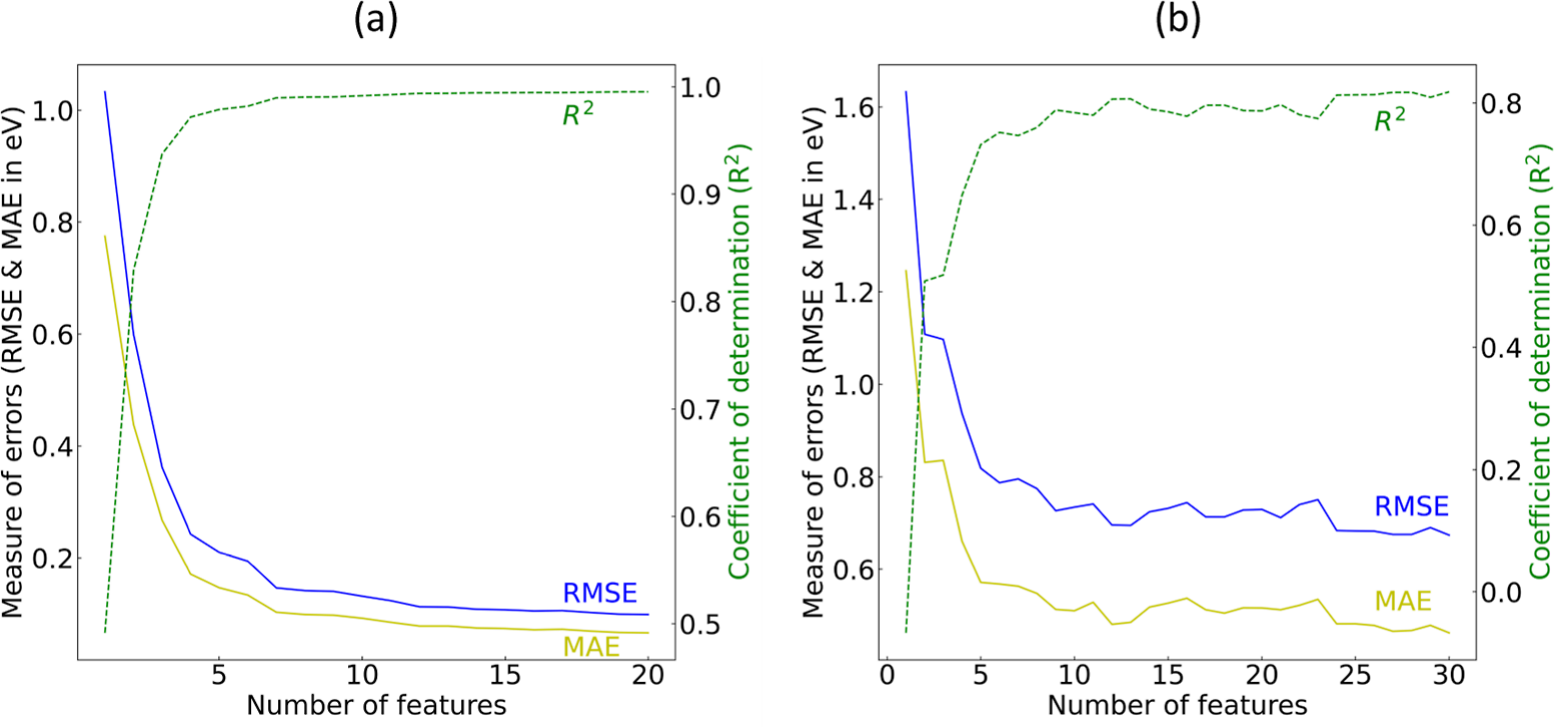
Gradient boosting feature selection (GBFS) result of the regression analysis of *E*_g_. Model performance of GBDTs on (a) the training set and (b) the validation set, where regression models are trained recursively with an increasing subset of features, beginning from the most relevant feature based on the realized total loss reduction.

The regression analysis considered 3896 compounds (*E*_g_ > 0), with 2458 materials of unique chemical compositions and a train-to-test split ratio of 4:1. In common with the classification problem, we employed only composition-based descriptors and processed the features via the GBFS workflow. This led to the selection of 46 features as the final subset of features among more than 820 exploratory features. The performance of the regression models during feature selection, on the training set and the validation set, is shown in Figure 6. The performance metrics of interest are *R*^2^, MAE, and RMSE. For both the training and the validation sets, the performance metrics plateaued before the first ca. 30 features, with a relatively worse performance on the out-of-sample validation set as anticipated. Subsequently, we performed the aforementioned statistical feature analyses and engineering, multicollinearity reduction, and permutation-importance analysis (Supporting Information 7), RFE (Supporting Information 8), and Bayesian optimization of the final regression model (Supporting Information 9), and we evaluated the total loss reduction achieved by the selected 46 features (Supporting Information 10). An independent feature analysis was conducted using the SHAP framework, which is in agreement with the features selected by the GBFS workflow (Supporting Information 11).

### 3.3 Multifidelity Modeling

#### 3.3.1 Multifidelity Model

**Figure 7:**
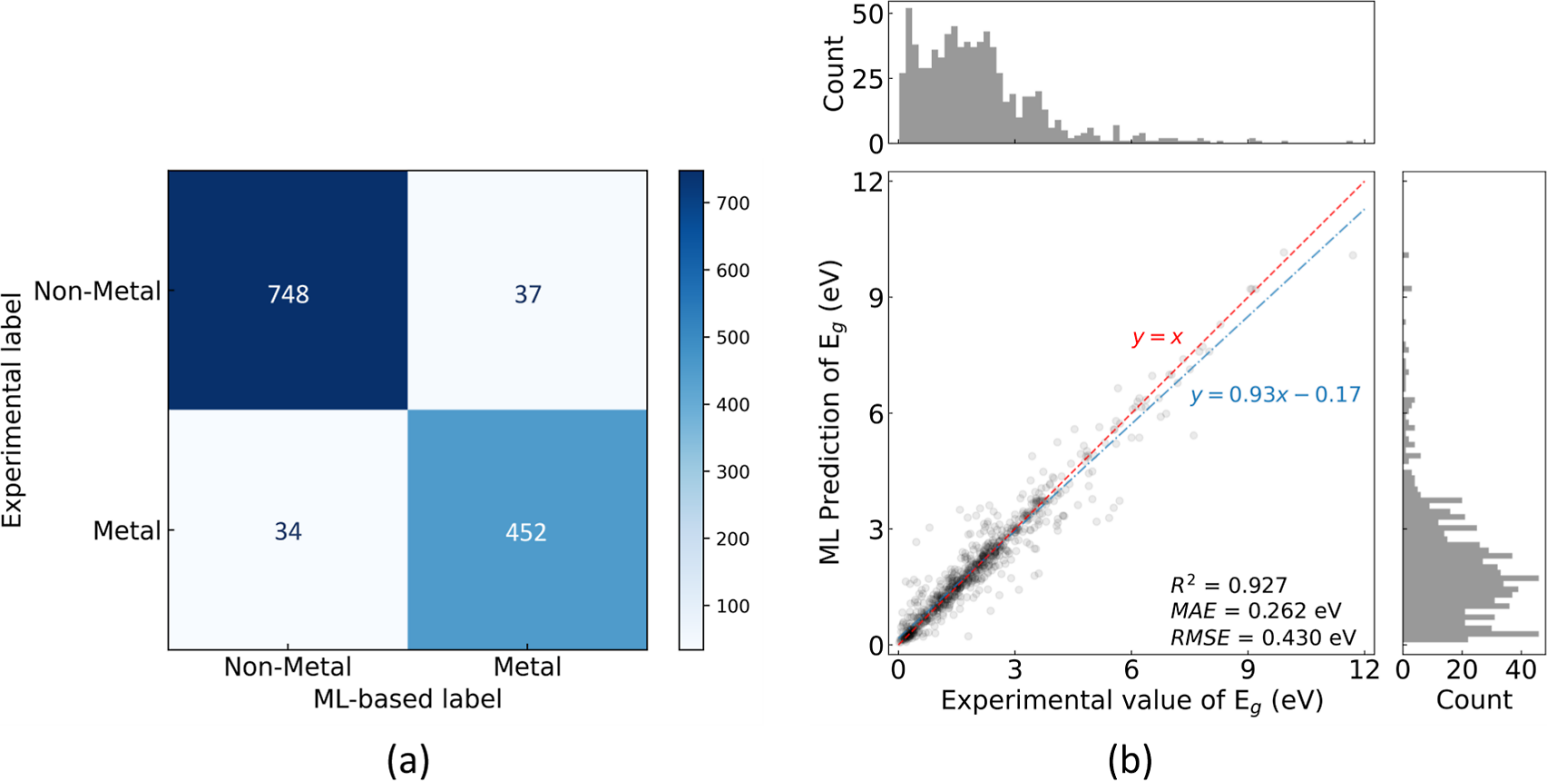
Multifidelity model performance against experimental measurements and DFT-based computed results for the (a) classification of materials by metallicity and (b) regression analysis of *E*_g_ in the test set, where the classification model was trained with 22 features and the regression model was trained on 25 features, both selected via the GBFS workflow. For the classification process, a macro-weighted AUC-ROC of 0.987, AP of 0.982, F1-score of 0.941, and a balanced accuracy of 0.941 were achieved.

Another direction explored in this research was to develop a multifidelity model using 154,718 DFT results from the MP, with 105,584 unique chemical compositions. An auxiliary model was trained on these chemical compositions of materials for the classification process and on 69,219 chemical compositions with *E*_g_ > 0.35 eV for the regression process, both using the GBFS workflow and a train-to-test split ratio of 4:1 (see Supporting Information 12 for the performance of the auxiliary models). An energy cutoff of 0.35 eV was applied to the DFT data set in order to mitigate inherent errors in DFT calculations of *E*_g_. We anticipated that the multifidelity approach could account for the lack of wide band gap semiconductors. However, we observed a comparable performance to that of the models that had been constructed without the multifidelity strategy. The results are listed in Figure 7. Closer examination of the feature interactions showed that the incorporation of DFT-based auxiliary models led to a smaller final subset of features selected via the GBFS workflow: 22 features for the classification and 25 features for the regression. These correspond to a reduction of 5 features in the former and a reduction of 21 features for the latter. Moreover, DFT-aware features are among the top three in the feature relevance ranking, which is based on the loss reduction realized when training the predictive models (Supporting Information 13). This demonstrates the benefit associated with such a modeling approach, which has gained popularity within computational material science.

It is crucial to distinguish our work from the multifidelity ML modeling approach conducted by Pilania et al.^29^ Their methodology can be classified as multifidelity because it incorporates calculations on 600 chemical compounds using different functionals for each compound, which have varying levels of exchange correlation within DFT. This entails the amalgamation of low- and high-fidelity DFT calculations, enabling cost-effective predictions at a higher fidelity level. Given the multifidelity modeling approach in our work arises from the fact that we have used computational and experimental data sources as input, a comparison of our work with theirs presents challenges, as their exclusive focus on computational data in the context of multifidelity ML models differs from our approach. Nevertheless, the use of a multifidelity approach in their work yielded improvements, while our study demonstrated a singular effect—a substantial reduction in the feature space. Despite being the sole observed outcome, this reduction remains a crucial and noteworthy result, as it can lead to a reduction in the feature space by up to ca. 46%; this renders many of the essential features identified in Section 3.2 no longer necessary.

It is unsurprising to encounter this difference, considering the limited data set of 600 chemical compounds in the study by Pilania et al.^29^ In such cases, the amalgamation of multiple data sources in the training process would prove advantageous. This is because the augmented training data for the multifidelity model can either facilitate the exploration of a larger chemical space, often mitigating the necessity for extreme extrapolation of learned relationships into unseen territories, or strengthen existing relationships with greater statistical certainty.

To demonstrate this, we employed a pseudocomparison multifidelity modeling approach utilizing only 600 experimental measurements that were randomly selected, in conjunction with ca. 150,000 DFT calculations. More specifically, we conducted two separate regression analyses: the first involved 600 experimental measurements, and the second utilized the same 600 experimental measurements alongside ca. 150,000 DFT calculations, employing a multifidelity modeling strategy. In the former scenario, we attained an *R*^2^ of 0.80, MAE of 0.50 eV, and RMSE of 0.77 eV. Conversely, in the latter scenario, we achieved an *R*^2^ of 0.86, MAE of 0.44 eV, and RMSE of 0.65 eV. As anticipated, the implementation of the multifidelity modeling strategy led to improvements in the quantified statistical figures-of-merit compared to a standard regression model; this is despite the recognized errors inherent in DFT calculations. Our analysis suggests a trade-off between the broader chemical coverage achieved by incorporating DFT calculations and the inherent uncertainties associated with them. Notably, the latter serves as a limiting factor in this study.

#### 3.3.2 Another Experimental Data Set

**Figure 8:**
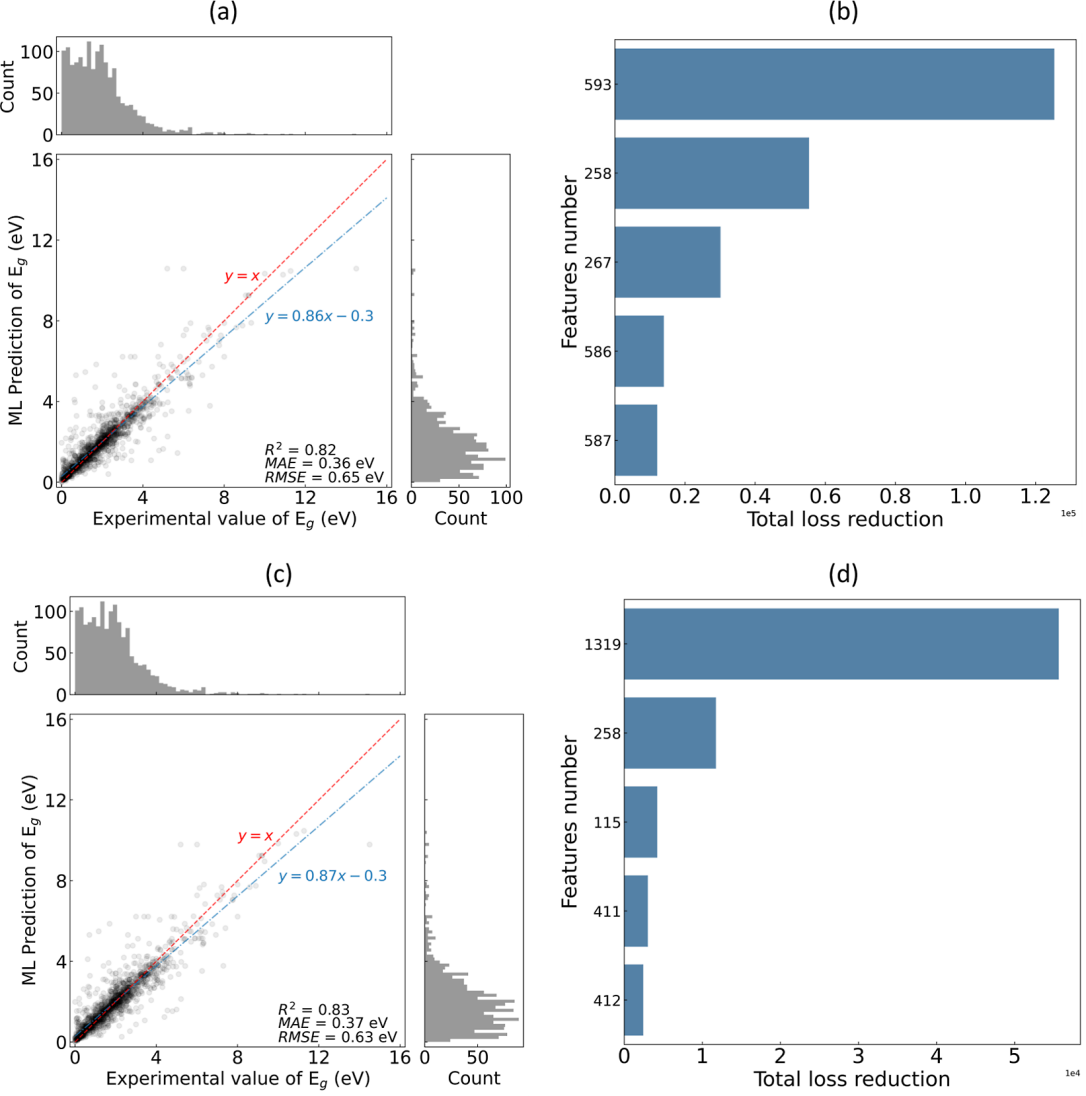
Regression analysis of *E*_g_ in the test set against (a) experimental measurements with 42 features, with the 5 most relevant features shown in (b), and against (c) experimental measurements and DFT calculations (i.e., using the multifidelity modeling approach) with 16 features, with the five most relevant features shown in (d). The features were selected via the GBFS workflow. Feature number 1319 corresponds to the DFT-based prediction of *E*_g_ values, which realized the largest total loss reduction. See *feature*_*list*.*csv* for the full list of feature names.

We extended our regression analysis to another set of experimental measurements, namely, those reported by Kiselyova et al.^64^ Their data set consists of 7588 chemical compositions of materials, with 3233 unique chemical compositions. The result with and without implementing a multifidelity modeling strategy is shown in Figure 8. In common with the findings noted above, we find that the model performances are comparable to each other. The use of the multifidelity approach resulted in a reduction of 26 features, with a predominant portion of the total loss reduction being attributed to the DFT-based prediction of *E*_g_ values. We infer that while DFT calculations facilitate the creation of less complex models in terms of the number of input features, they do not necessarily improve the model accuracy, possibly because of the inherent limitations associated with these calculations.

Moreover, this experimental data set is used to assess the predictions made on the chemical compositions within Pearson’s Crystal Structure Database (94,095) and the MP (105,583). A sample of 60 chemical compositions, previously unseen by the model, was randomly chosen, and the predictions were compared against the experimental measurements. The results are summarized in Table 3. As anticipated, the model has a tendency to underestimate the experimental *E*_g_ values, with 37 and 39 (out of 60) chemical compositions exhibiting a negative percentage difference from the mean and from the median *E*_g_ values, respectively. The average absolute percentage differences from the mean and from the median are ca. 15 and 13%, respectively. Notably, a lower absolute percentage difference is observed when considering the median *E*_g_ value for specific compounds such as BaCu_2_GeS_4_, PPdS, KP(HO_2_)_2_, FeS_2_, and As. This suggests that a lower deviation can be realized when considering the median as the measure of central tendency in the presence of multiple experimental measurements, as outliers exert a relatively minimal effect on the median of a given data set compared to the mean. These predicted *E*_g_ values are included as Supporting Information, potentially serving as a valuable resource for researchers engaged in the study of inorganic materials and their band gaps.

**Table 3:** Examples of Input Chemical Composition and the Corresponding Prediction of Band Gap (*E*_g_), Sorted by the Absolute Percentage Difference from the Mean Experimental Values in Ascending Order^a^

composition	experimental *E*_*g*_ (eV)					pred. *E*_*g*_ (eV)	% diff. from mean	% diff. from median
	*n*	min	max	mean	median			
Ga_2_(TeO_3_)_3_	2	4.14	4.15	4.15	4.15	4.11	–1.0	–1.0
Hg_3_(SI)_2_	1	2.25	2.25	2.25	2.25	2.22	–1.3	–1.3
BaGeS_3_	1	2.46	2.46	2.46	2.46	2.49	1.2	1.2
HgCl	3	2.84	3.80	3.38	3.50	3.43	1.5	–2.0
K_3_Th_2_Cu_3_S_7_	1	2.49	2.49	2.49	2.49	2.45	–1.6	–1.6
Bi_2_SO_2_	2	1.12	1.50	1.31	1.31	1.33	1.5	1.5
K_2_Hg_3_(GeS_4_)_2_	2	2.64	2.70	2.67	2.67	2.71	1.5	1.5
La_3_Mo_4_O_16_F	1	3.70	3.70	3.70	3.70	3.64	–1.6	–1.6
Ba_3_Cd(SnS_4_)_2_	1	2.75	2.75	2.75	2.75	2.80	1.8	1.8
Cs_2_Ba_3_(P_2_O_7_)_2_	2	5.06	6.31	5.69	5.69	5.79	1.8	1.8
Ga_2_PbS_4_	4	2.38	2.55	2.46	2.46	2.41	–2.0	–2.0
La_4_Fe(SbS_5_)_2_	1	1.00	1.00	1.00	1.00	1.03	3.0	3.0
Zn(InTe_2_)_2_	7	1.13	1.95	1.74	1.87	1.68	–3.4	–10.2
BaCu_2_GeS_4_	8	2.29	2.49	2.43	2.47	2.53	4.1	2.4
Li_2_TeMoO_6_	1	3.50	3.50	3.50	3.50	3.34	–4.6	–4.6
PHPb_2_N_2_O_9_	1	3.81	3.81	3.81	3.81	3.62	–5.0	–5.0
NbIn(TeO_4_)_2_	1	3.50	3.50	3.50	3.50	3.32	–5.1	–5.1
In_5_AgS_8_	3	1.60	1.79	1.72	1.76	1.63	–5.2	–7.4
Ba_2_DyGaSe_5_	2	2.35	2.35	2.35	2.35	2.22	–5.5	–5.5
Na_4_Mg(SiSe_3_)_2_	1	2.85	2.85	2.85	2.85	2.69	–5.6	–5.6
Na_2_Cd(GeS_3_)_2_	3	2.57	3.21	2.79	2.60	2.96	6.1	13.8
Ba_2_ErGaSe_5_	2	1.95	1.95	1.95	1.95	2.07	6.2	6.2
InHg_7_S_6_Cl_5_	1	2.54	2.54	2.54	2.54	2.37	–6.7	–6.7
BaAl_4_S_7_	1	3.74	3.74	3.74	3.74	3.48	–7.0	–7.0
Rb_2_Cd_3_(B_4_O_7_)_4_	1	4.76	4.76	4.76	4.76	4.42	–7.1	–7.1
Cd(GaSe_2_)_2_	18	2.10	2.75	2.39	2.41	2.56	7.1	6.2
KThCuS_3_	1	2.95	2.95	2.95	2.95	2.72	–7.8	–7.8
Sb_8_I_2_O_11_	5	2.72	2.72	2.72	2.72	2.50	–8.1	–8.1
KCu_2_BiS_3_	1	1.29	1.29	1.29	1.29	1.40	8.5	8.5
K_2_Mn(SnSe_3_)_2_	1	2.00	2.00	2.00	2.00	1.82	–9.0	–9.0
Rb_2_VAgS_4_	1	1.83	1.83	1.83	1.83	2.00	9.3	9.3
PrMoO_4_F	1	3.64	3.64	3.64	3.64	3.30	–9.3	–9.3
ZnSi(AgS_2_)_2_	1	3.28	3.28	3.28	3.28	2.96	–9.8	–9.8
K_2_Sn_2_Hg_3_S_8_	2	2.40	2.50	2.45	2.45	2.19	–10.6	–10.6
PPdS	12	0.70	1.40	1.16	1.38	1.30	12.1	–5.8
RbH_2_(IO_3_)_3_	2	4.07	5.08	4.58	4.58	4.00	–12.7	–12.7
In_4_Bi_3_S_10_	1	1.42	1.42	1.42	1.42	1.24	–12.7	–12.7
ZnCu_2_SiS_4_	3	3.00	3.25	3.17	3.25	2.75	–13.2	–15.4
Te_3_As_2_	28	0.48	1.88	1.22	1.35	1.40	14.8	3.7
NbInBi_2_O_7_	1	2.70	2.70	2.70	2.70	2.30	–14.8	–14.8
InCuGeSe_4_	1	1.30	1.30	1.30	1.30	1.50	15.4	15.4
BaBiBS_4_	1	2.34	2.34	2.34	2.34	1.98	–15.4	–15.4
NaSc(SeO_3_)_2_	1	5.50	5.50	5.50	5.50	4.56	–17.1	–17.1
RbBaPS_4_	1	3.30	3.30	3.30	3.30	2.70	–18.2	–18.2
Ba_3_Er_2_(PS_4_)_4_	1	3.30	3.30	3.30	3.30	2.69	–18.5	–18.5
SbPbIO_2_	1	2.48	2.48	2.48	2.48	1.98	–20.2	–20.2
Zn_3_(PS_4_)_2_	2	3.07	3.19	3.13	3.13	2.49	–20.4	–20.4
AgBi(PSe_3_)_2_	1	1.40	1.40	1.40	1.40	1.11	–20.7	–20.7
NaB_5_(H_2_O_5_)_2_	2	5.61	6.13	5.87	5.87	7.13	21.5	21.5
Cs_2_Mg_2_(WO_4_)_3_	1	4.53	4.53	4.53	4.53	3.46	–23.6	–23.6
KP(HO_2_)_2_	3	3.20	7.00	5.72	6.95	7.09	24.0	2.0
Ti(Bi_3_O_5_)_4_	1	3.09	3.09	3.09	3.09	2.33	–24.6	–24.6
LiZnPS_4_	1	3.44	3.44	3.44	3.44	2.57	–25.3	–25.3
NaYb(PS_3_)_2_	1	1.85	1.85	1.85	1.85	2.37	28.1	28.1
FeS_2_	9	0.02	1.25	0.83	0.92	1.17	41.0	27.2
Bi_4_Pb_7_Se_13_	4	0.23	0.29	0.26	0.26	0.14	–46.2	–46.2
Hg_8_Bi_3_As_4_Cl_13_	1	4.30	4.30	4.30	4.30	2.04	–52.6	–52.6
SrBe_2_(BO_3_)_2_	1	4.69	4.69	4.69	4.69	7.32	56.1	56.1
Ba_8_U_2_PdSe_16_	2	0.18	1.60	0.89	0.89	1.43	60.7	60.7
As	10	0.17	1.25	0.80	1.14	1.38	72.5	21.1

a Data from ref (64). Here, *n* is the number of experimental values sampled; min and max represents their range; mean and median are their corresponding descriptive statistics.

## 4 Conclusions

This study has employed a ML-based feature selection and statistical feature analysis workflow to train predictive models that classify materials by their metallicity and predict their band gap (*E*_g_). Our feature-selection workflow integrates a distributed gradient boosting framework, in conjunction with exploratory data and statistical analyses and multicollinearity treatments, to identify and select a subset of features that is highly relevant to the target variable or class within a complex feature space; this affords minimal feature redundancy and maximal relevance to the target variable or classes. Gradient boosting trees are subsequently trained with the selected features, which are solely based on the chemical composition of a material. The classification model realized a macro-averaged F1-score of 0.940, AP of 0.977, and AUC-ROC of 0.985, while the regression model achieved an MAE of 0.246, RMSE of 0.402, and *R*^2^ of 0.937. The results are superior to high-throughput DFT calculations that employ a PBE functional while being either superior to or comparable to complex algorithms reported in the literature. This exemplifies the efficacy of our modeling approach and highlights the importance of thorough feature analysis and judicious selection over merely complex modeling. We further explored the multifidelity modeling strategy and found that such an approach can reduce the number features required to train a model. We applied our models to chemical compositions in Pearson’s Crystal Structure Database (94,095) and the MP (105,583). The results are made available as a part of the Supporting Information, serving as a resource for researchers in the development of novel inorganic materials.

## Data Availability

We have made the code for the feature selection, statistical analyses, multicollinearity reduction, RFE, and Bayesian optimization available at https://github.com/Songyosk/BGML. The data sets used in this work are available from the MP v2022.10.28^31,32^ and the Matbench test suite v0.1.^57^ The aggregated nonmetal data and our model predictions are provided in *bandgap*_*data*.*xlsx* as a part of the Supporting Information.

## Supporting Information Available

Class-probability distributions, hierarchical cluster and permutation importance analyses, recursive feature elimination, convergence, partial dependence, and evaluation plots obtained during Bayesian optimization, feature-relevance ranking, SHAP analysis, for both the regression and the classification problem, and band gap predictions against DFT calculations and the feature-relevance score for the multifidelity models (PDF)
Full list of feature names and their corresponding feature numbers (XLSX)
Aggregated nonmetal data and model predictions (XLSX)
