# Supplementary material for: Automatic Prediction of Band Gaps of Inorganic Materials Using a Gradient Boosted and Statistical Feature Selection Workflow
Source: J Chem Inf Model. 2024 Feb 6;64(4):1187–200. doi: 10.1021/acs.jcim.3c01897 (PMC10900294; doi:10.1021/acs.jcim.3c01897)
Supplement: Supplementary file 1 — ci3c01897_si_001.pdf [file ci3c01897_si_001.pdf]

Supporting Information

Automatic Prediction of Band Gaps of Inorganic Materials using a Gradient Boosted and Statistical Feature Selection Workflow

Son Gyo Jung[1,2,3], Guwon Jung[1,3,4], Jacqueline M. Cole[1,2,3,*]

[1]*Cavendish Laboratory, Department of Physics, University of Cambridge, J. J. Thomson Avenue, Cambridge, CB3 0HE, UK*

[2]*ISIS Neutron and Muon Source, STFC Rutherford Appleton Laboratory, Harwell Science and Innovation Campus, Didcot, Oxfordshire, OX11 0QX, UK*

[3]*Research Complex at Harwell, Rutherford Appleton Laboratory, Harwell Science and Innovation Campus, Didcot, Oxfordshire, OX11 0FA, UK*

[4]*Scientific Computing Department, STFC Rutherford Appleton Laboratory, Harwell Science and Innovation Campus, Didcot, Oxfordshire, OX11 0QX, UK*

[*]jmc61@cam.ac.uk

SI. 1 - Class-probability distributions

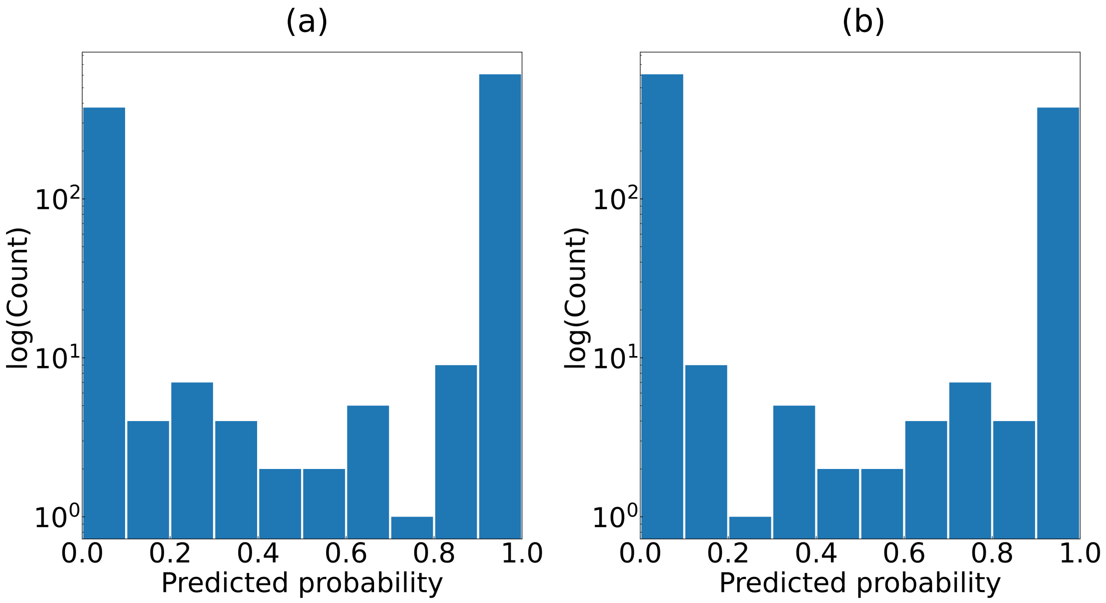

Figure S1: The predicted probability distribution of the final ML classifier for (a) non-metal and (b) metal in logarithmic scale. The output class-probabilities of the classifier are predominantly around zero and one, illustrating almost a binary outcome and demonstrates that the model is highly discriminative towards the target classes.

SI. 2 - Hierarchical cluster analysis & permutation importance for classification

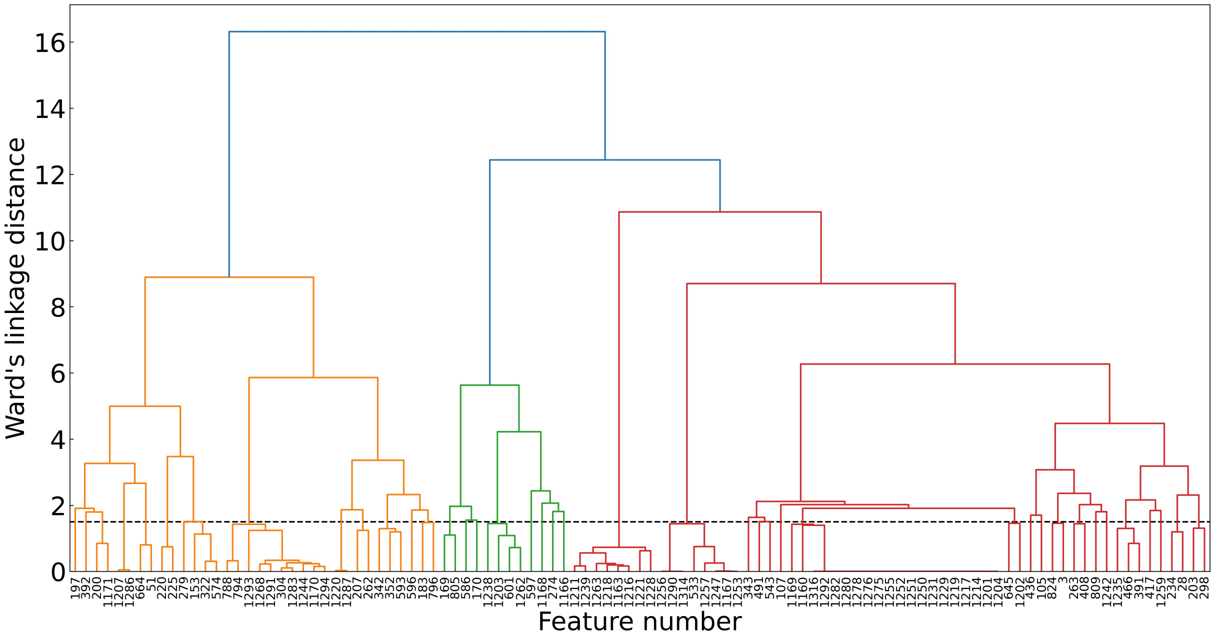

Figure S2.1: Multicollinearity reduction - the dendrogram of the hierarchical agglomerative clustering using the remaining 105 features after performing the correlation analysis. The dashed horizontal line in black represents the distance threshold of 1.5 units of Ward's linkage distance. See *feature_list.csv* for the full list of feature names.

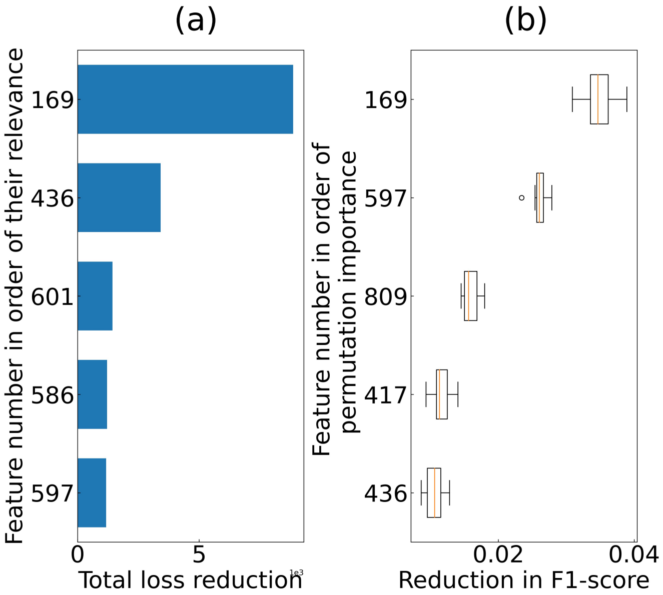

Figure S2.2: (a) Results of the feature-relevance analysis prior to the recursive feature elimination stage, showing the five most relevant features. (b) The permutation feature-importance plot for the classification of material by metallicity. See *feature_list.csv* for the full list of feature names.

SI. 3 - Recursive feature elimination for classification

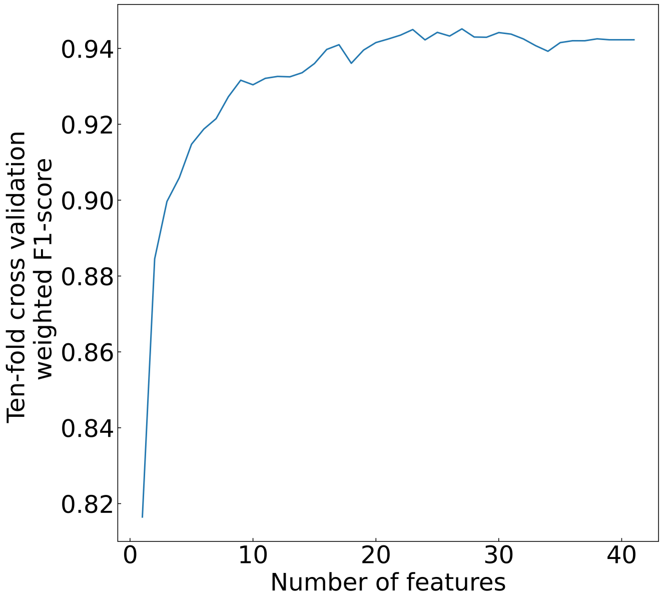

Figure S3: The ten-fold RFE result using the weighted F1-score as the metric.

SI. 4 - Bayesian optimization for classification

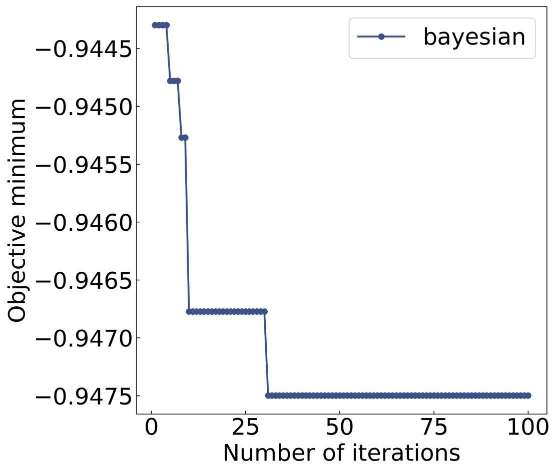

Figure S4.1: The convergence plot of four different optimization techniques for the regression.

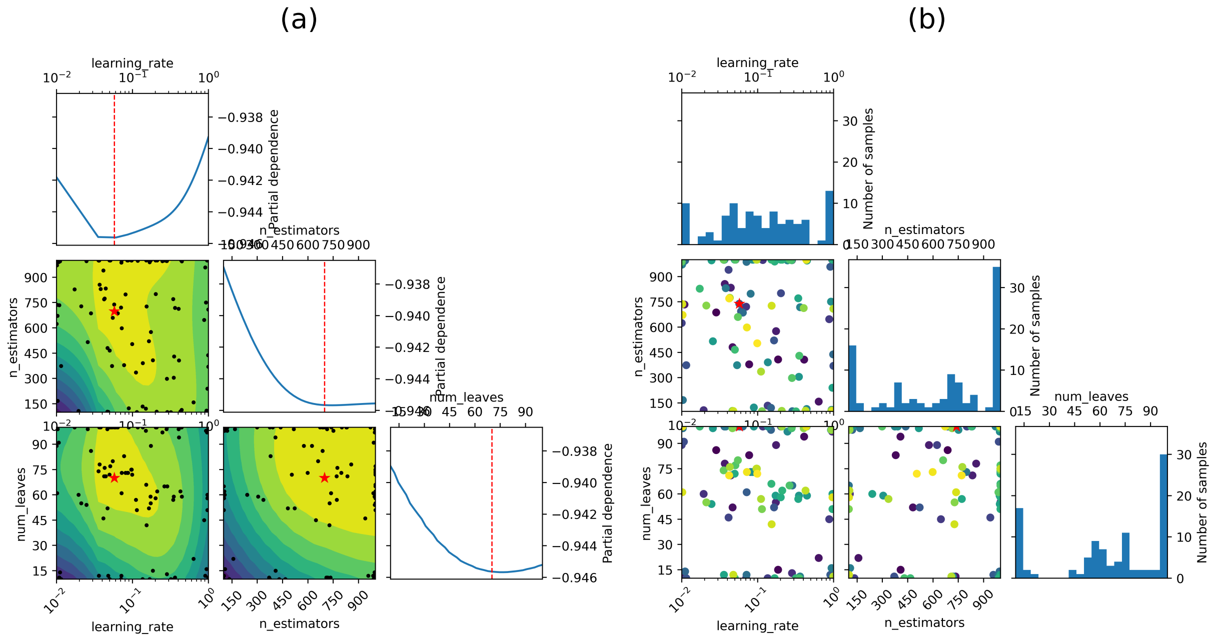

Figure S4.2: The Bayesian optimization results of the final classification model using the training data, where (a) is the partial dependence plot and (b) is the evaluation plot. The red stars indicate the values of the hyperparameters that achieved the lowest value of the objective function. The approximate position of the objective minimum is indicated by the dashed vertical lines in red.

SI. 5 - Feature-relevance ranking of final classifier

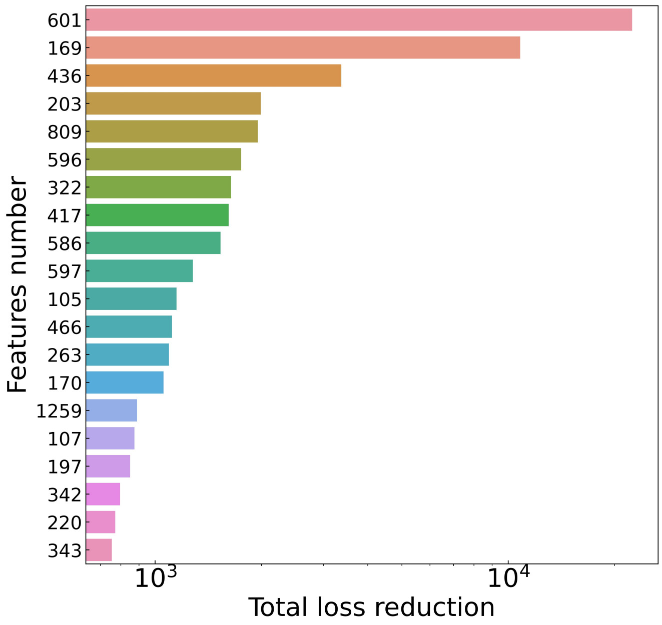

Figure S5: The final 20 most relevant features selected for the classification of materials by metallicity and the realized total loss reduction. See *feature_list.csv* for the full list of feature names.

SI. 6 - SHAP analysis for classification

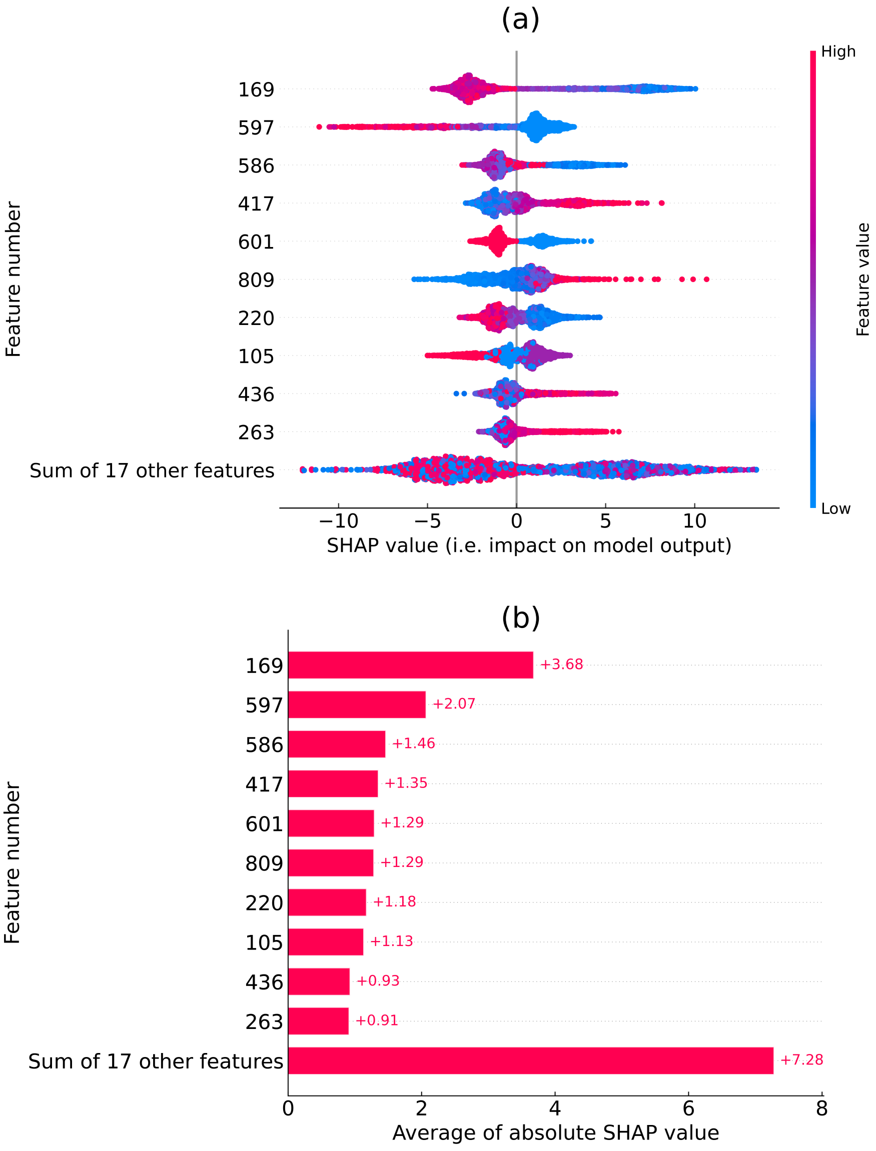

Figure S6: The results based on the SHAP framework: (a) the average contribution (i.e. the mean absolute SHAP value) of ten features that are identified as having the most contribution to the model output. A positive SHAP value indicates a positive contribution to the classification of materials by their metallicity. (b) The beeswarm plot illustrates the impact of these features on the model output by plotting each instance as a single data point together with the SHAP value on the x-axis, where the y-axis is consistent with (a). The color scheme corresponds to the original feature value and the broadening shows the density of instances (cf. density plot). See *feature_list.csv* for the full list of feature names.

SI. 7 - Hierarchical cluster analysis & permutation importance for regression analysis

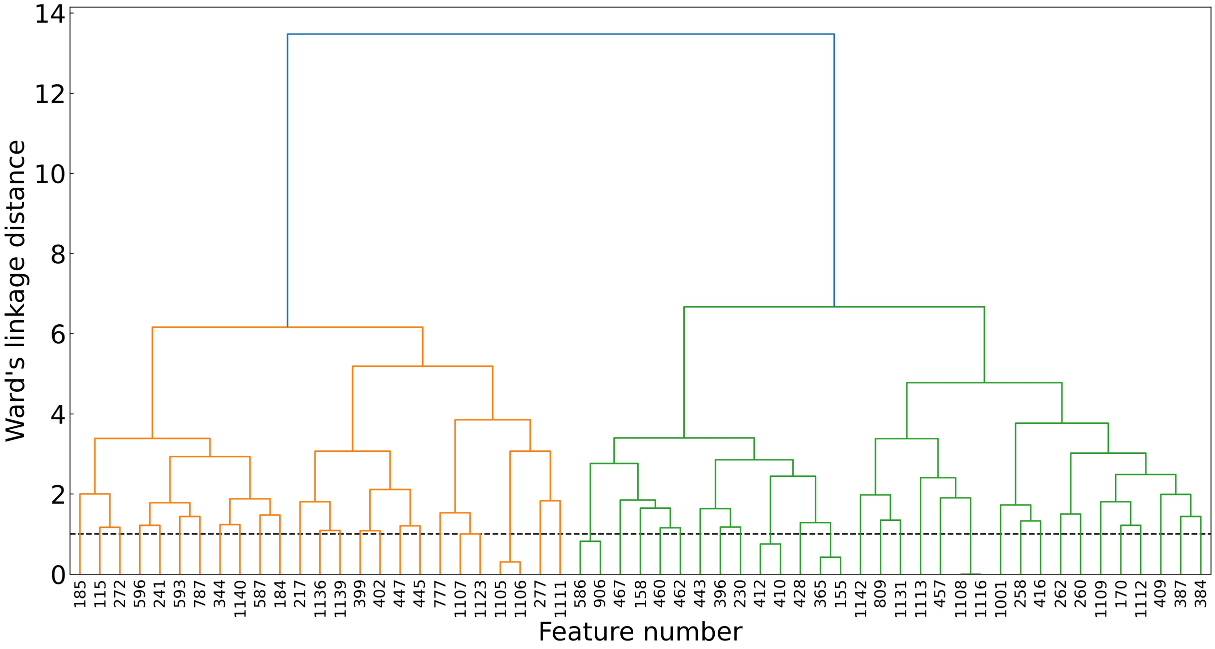

Figure S7.1: Multicollinearity reduction - the dendrogram of the hierarchical agglomerative clustering using the remaining 57 features after performing the correlation analysis. The dashed horizontal line in black represents the distance threshold of an unit of Ward's linkage distance. See $feature\_list.csv$ for the full list of feature names.

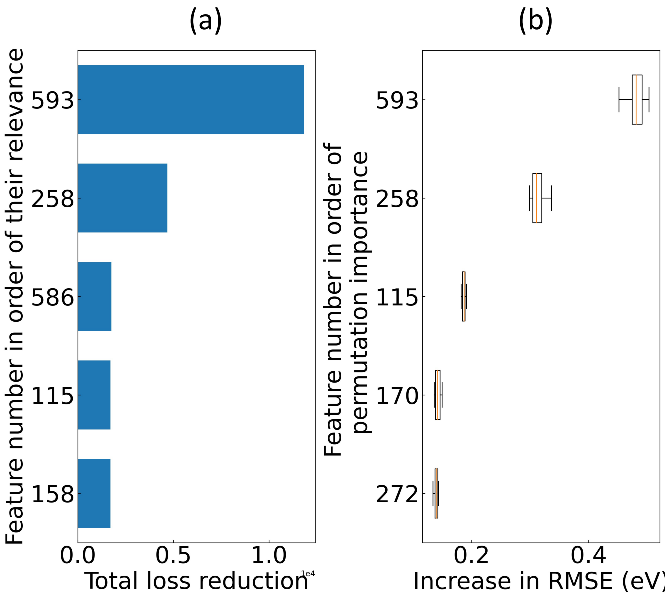

Figure S7.2: (a) Results of the feature-relevance analysis prior to the recursive feature elimination stage, showing the five most relevant features. (b) The permutation feature-importance plot for the regression of band gap. See $feature\_list.csv$ for the full list of feature names.

SI. 8 - Recursive feature elimination for regression analysis

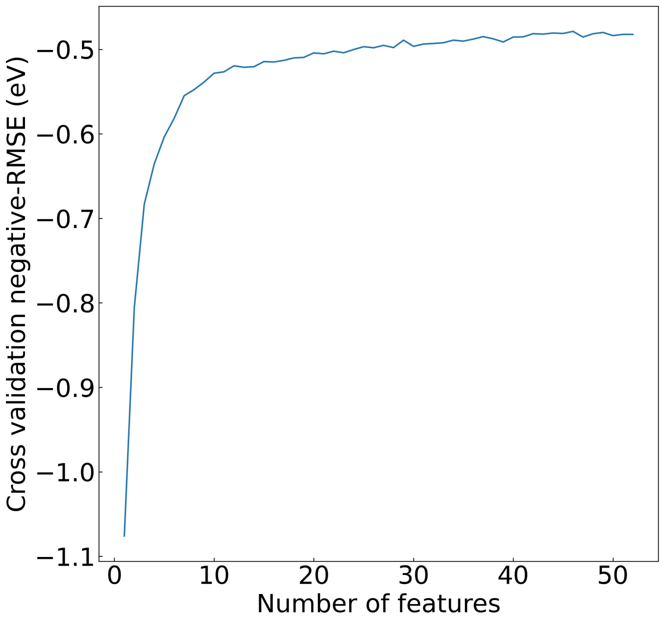

Figure S8: The ten-fold RFE result using the negative-RMSE as the metric.

SI. 9 - Bayesian optimization for regression analysis

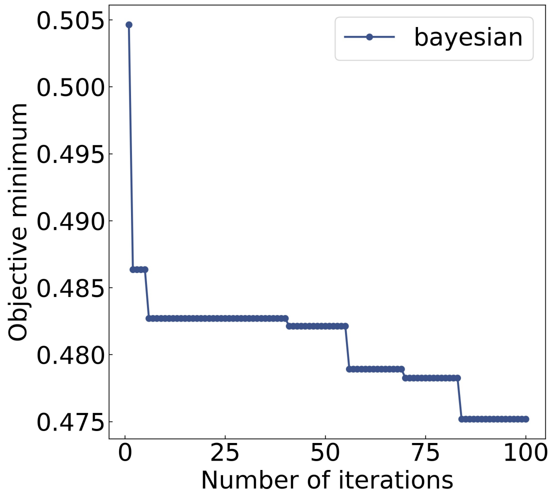

Figure S9.1: The convergence plot of four different optimization techniques for the regression model.

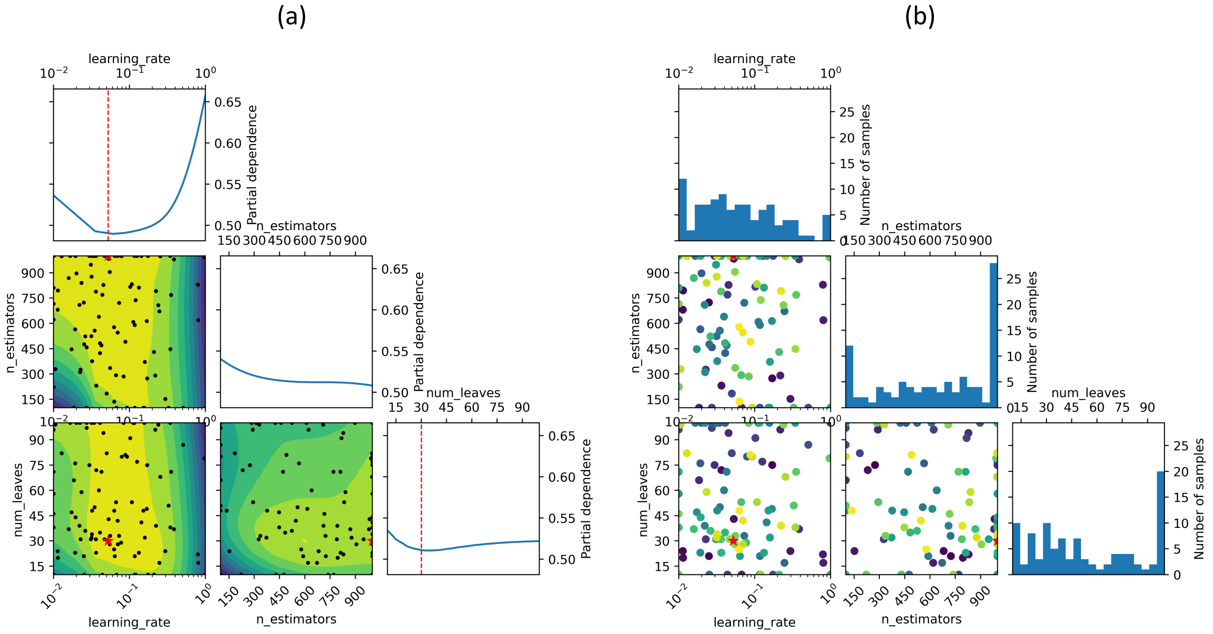

Figure S9.2: The Bayesian optimization results of the final regression model using the training data, where (a) is the partial dependence plot and (b) is the evaluation plot. The red stars indicate the values of the hyperparameters that achieved the lowest value of the objective function. The approximate position of the objective minimum is indicated by the dashed vertical lines in red.

SI. 10 - Feature-relevance ranking of final regression model.

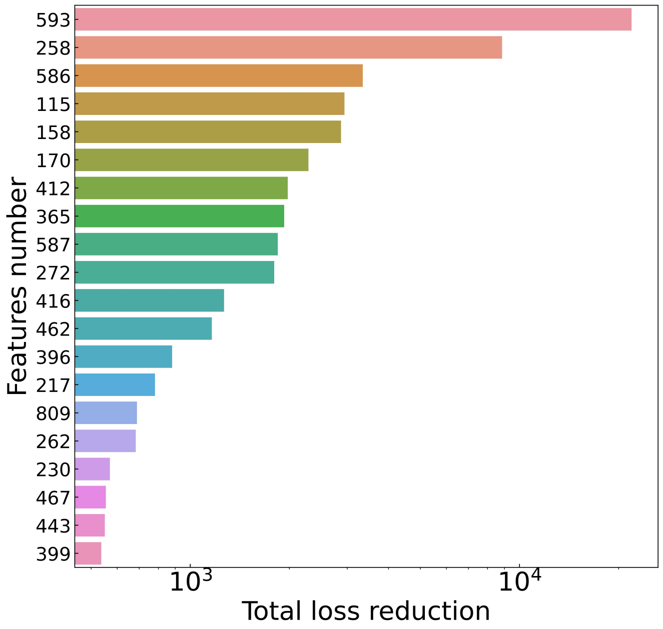

Figure S10: The final 20 most relevant features selected for the regression analysis of band gap and the realized total loss reduction. See *feature_list.csv* for the full list of feature names.

SI. 11 - SHAP analysis for regression problem

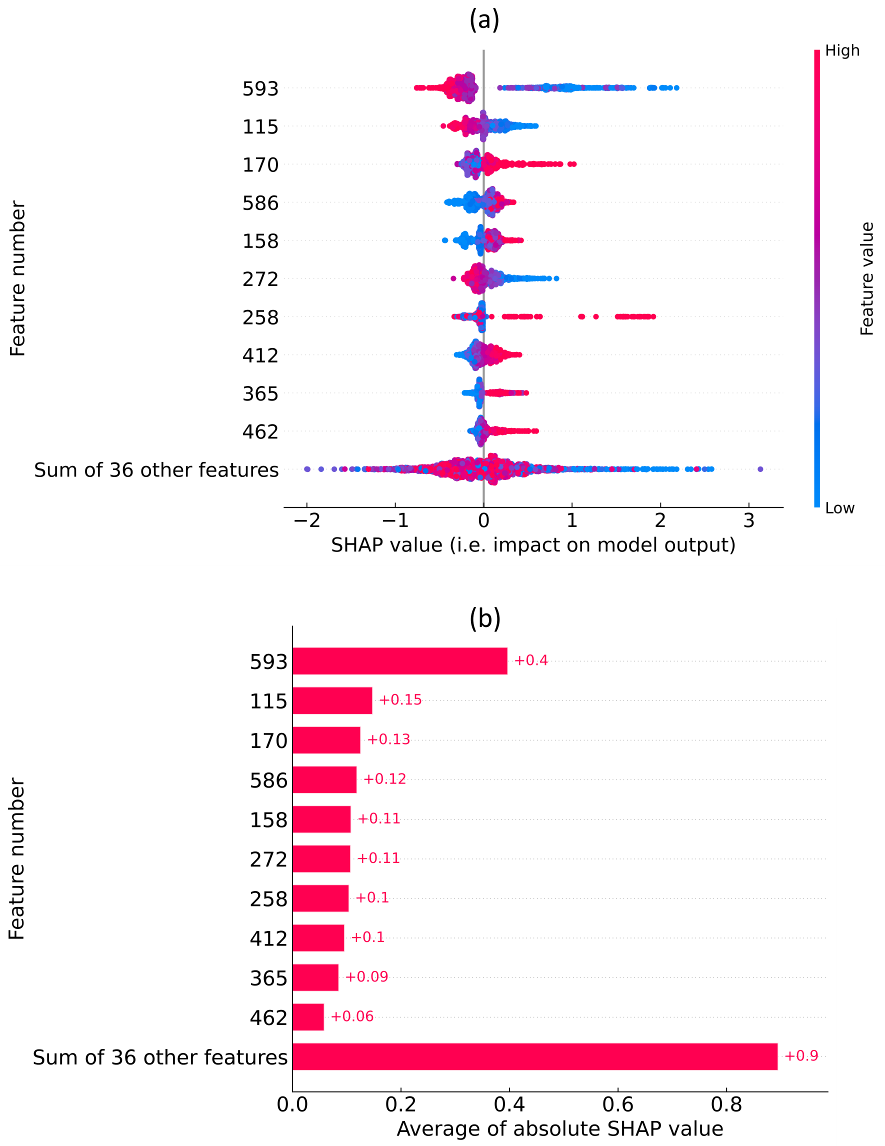

Figure S11: The results based on the SHAP framework: (a) the average contribution (i.e. the mean absolute SHAP value) of ten features that are identified as having the most contribution to the model output. A positive SHAP value indicates a positive contribution to the prediction of band gap. (b) The beeswarm plot illustrates the impact of these features on the model output by plotting each instance as a single data point together with the SHAP value on the x-axis, where the y-axis is consistent with (a). The color scheme corresponds to the original feature value and the broadening shows the density of instances (cf. density plot). See *feature_list.csv* for the full list of feature names.

SI. 12 - Predictions against DFT calculations

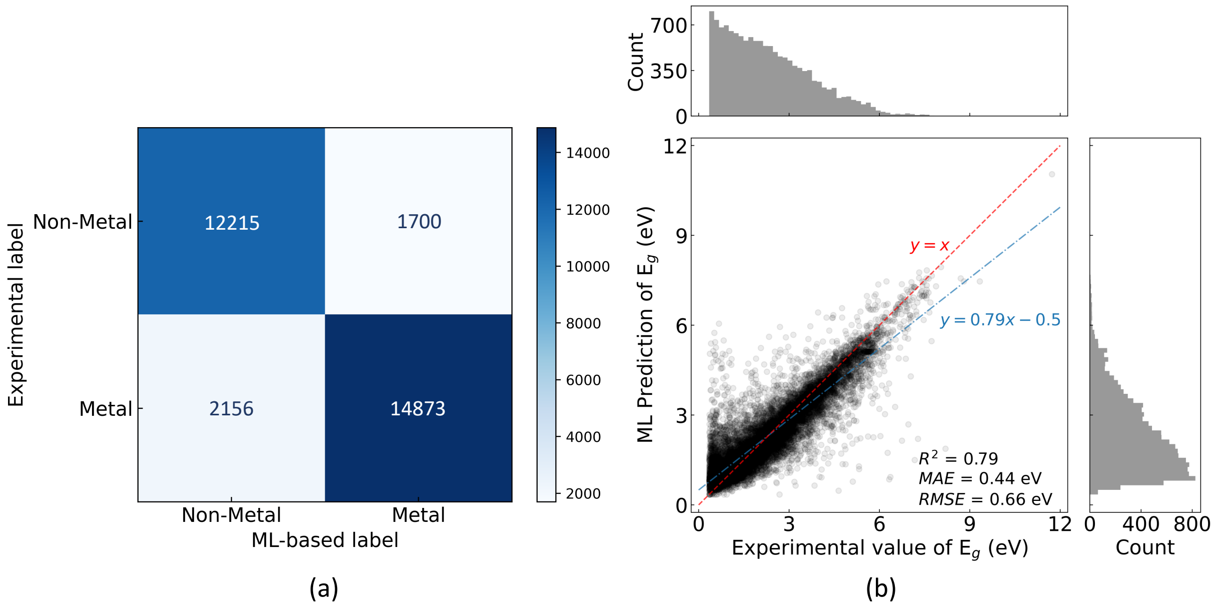

Figure S12: Bayesian-optimized model performance against DFT calculations (Materials Project) for the (a) classification of materials by metallicity and (b) regression analysis of E_g the test set, where the classification model was trained with 35 features and the regression model was trained on 44 features, both selected via the GBFS workflow. For the classification, a macro-weighted ROC-AUC of 0.949, AP of 0.962, F1-score of 0.874 and an balanced accuracy of 0.876 were achieved.

SI. 13 - Feature-relevance score for the multi-fidelity models

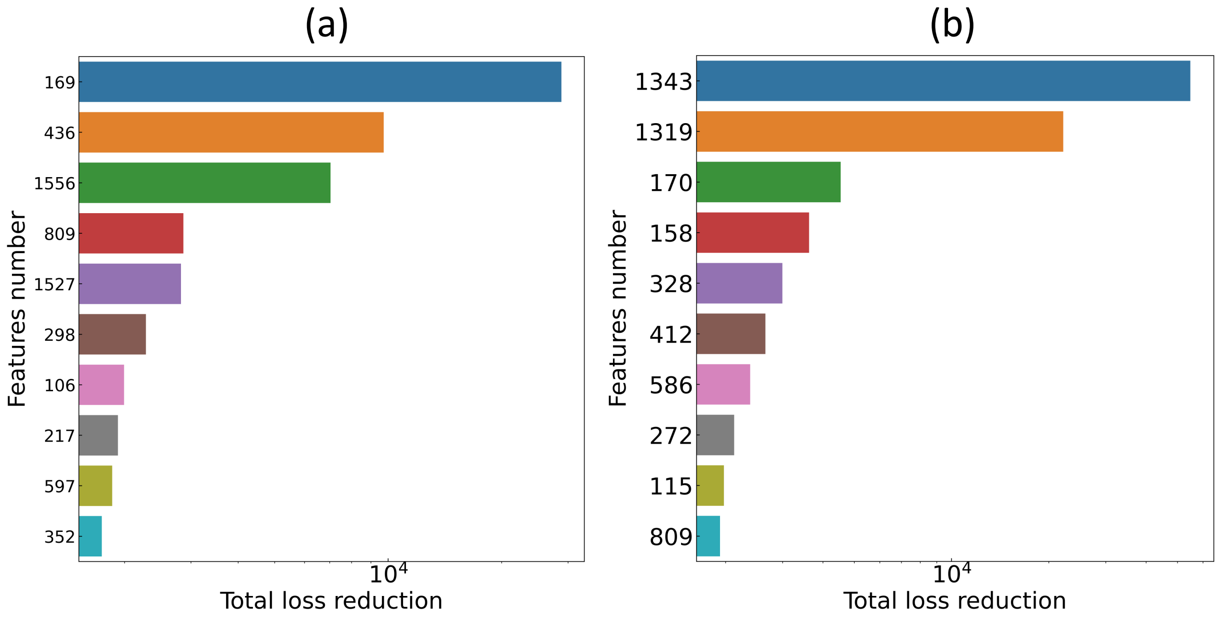

Figure S13: The total loss reduction realized by the features in the (a) classification and (b) regression analyses using the multi-fidelity strategy. See *feature_list.csv* for the full list of feature names.
